# Acoustic Modulation of Excitonic Complexes in hBN/WSe_2_/hBN Heterostructures

**DOI:** 10.1021/acs.nanolett.4c03301

**Published:** 2024-11-25

**Authors:** Marcos L. F. Gomes, Pedro W. Matrone, Alisson R. Cadore, Paulo V. Santos, Odilon D. D. Couto

**Affiliations:** † Universidade Estadual de Campinas, Instituto de Física Gleb Wataghin, 13083-859 Campinas, Brazil; ¶ Laboratório Nacional de Nanotecnologia, Centro Nacional de Pesquisa em Energia e Materiais, 13083-100 Campinas, Brazil; § Paul-Drude-Institut für Festkörperelektronik, Leibniz-Institut im Forschungsverbund Berlin e.V., Hausvogteiplatz 5-7, 10117 Berlin, Germany

**Keywords:** WSe_2_, van der Waals heterostructure, surface acoustic waves, biexciton, exciton, trion

## Abstract

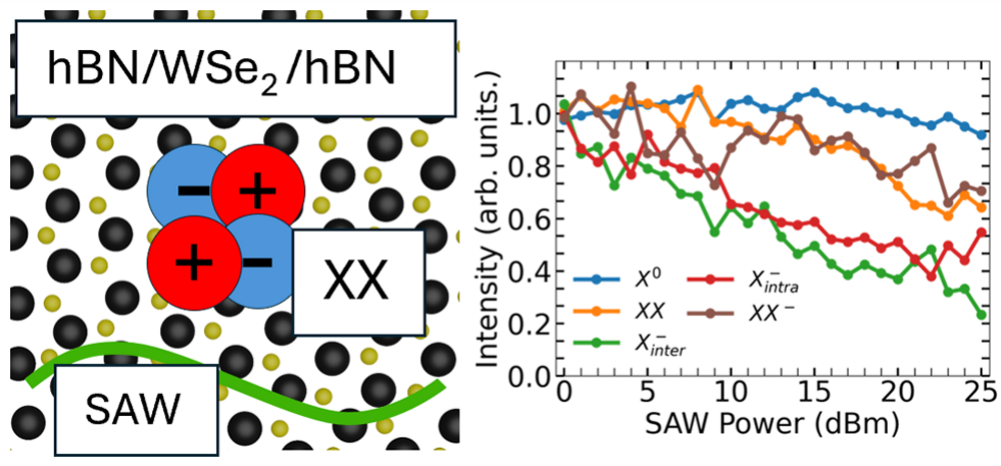

The interaction of high-frequency surface acoustic waves (SAWs) and excitons in van der Waals heterostructures (vdWHs) offers challenging opportunities to explore novel quantum effects and functionalities. We probe the interaction of neutral excitons, trions, and biexcitons with SAWs in a hBN/WSe_2_/hBN vdWH. We show that neutral excitons respond weakly to the SAW stimulus at 5 K. The remaining excitonic complexes, because of their lower binding energy or charged character, interact much more efficiently with the SAW piezoelectric field, particularly intra- and intervalley trions. At room temperature, the SAW can play a dual role (sometimes dissociating excitons and sometimes increasing the vdWH local doping density) which depends of the laser-induced photodoping of the vdWH prior to the SAW generation and the role of metastable energy states in the SAW-induced carrier dynamics. Our results shed light in the unexplored biexciton modulation with SAWs, important for 2D materials-based optoelectronic and energy harvesting devices.

The employment of surface acoustic waves (SAW) to controllably modify fundamental interactions in two-dimensional (2D) systems has attracted growing interest over the last years.^1^ The coupling of 2D materials with piezoelectric substrates has enabled experiments using propagating or stationary SAW modes to modulate the electronic properties and transport carriers in a variety of layered structures at relatively high frequencies. In graphene, the SAW-induced deformation has been used to control gauge fields acting on Dirac Fermions^2^ and to modulate Raman-phonon modes.^3^ The type-II lateral carrier separation induced by the SAW piezoelectric field has been used to suppress dark currents and improve the performance of few-layer MoS_2_^4^ and SnS_2_^5^ photodetectors as well as of van der Waals heterojunctions.^6^ SAWs have also been used to modulate the emission of hexagonal boron nitride (hBN) layers^7^ and the bandgap of ReS_2_ flakes.^8^

Transition metal dichalcogenide (TMD)-based 2D structures in their own right have attracted strong interest due to their symmetry-dependent excitonic quantum properties^9−11^ and applications.^12,13^ Many efforts have been spent to describe the interaction of SAWs and excitonic complexes in such type of structures.^14,15^ The role of the dielectric screening introduced by the piezoelectric substrate on the SAW-driven neutral and negatively charged exciton dynamics in nonencapsulated monolayer (1L) systems has been understood.^16,17^ Stationary modes of SAW cavities have been employed to modulate localized-state energy levels in nonencapsulated 1L-WSe_2_.^18^ In van der Waals heterostructures (vdWHs), exciton modulation at the SAW frequency and exciton transport using the SAW strain field in a hBN-encapsulated 1L-WSe_2_ at room temperature has been observed.^19^ Efficient interlayer exciton transport at 100 K in a bilayer WSe_2_ vdWH has also been demonstrated.^20^ However, a broad understanding of the interaction of SAWs and all the different kinds of excitonic complexes that can be optically generated in TMD structures is still lacking. The later is essential for envisioning more sophisticated applications which take advantage of the SAW itinerant phonons like integration with photonic circuits,^21^ quantum communication^22,23^ or signal processing at high frequencies.^24^

We probe the interaction of different excitonic complexes with SAWs under different temperature regimes in a high-quality hBN/1L-WSe_2_/hBN vdWH. At low temperatures, we detect efficient acoustic modulation of all excitonic complexes except the neutral exciton (X^0^), i.e., of the negatively charged intra (X_*intra*_^–^) and intervalley (X_*inter*_^–^) excitons, the neutral (XX) and charged (XX^–^) biexcitons, as well as localized states. We show that the increase in carrier mobility achieved by the hBN encapsulation makes the response to the acoustic stimulus faster in comparison to nonencapsulated monolayers, which is demonstrated by a fast on/off rate of photoluminescence (PL) quenching induced by the SAW piezoelectric field. On the other hand, the hBN underneath the 1L-WSe_2_ diminishes the strong dielectric screening associated with the LiNbO_3_ substrate employed for SAW excitation,^16,17^ making the interaction of X^0^ with the SAW less effective at low temperatures. The remaining excitonic complexes, however, interact strongly with the SAW piezoelectric field, mainly X_*intra*_^–^ and X_*inter*_^–^ which have an extra electron and lower binding energies. As the temperature increases, the thermal energy increases the response of X^0^ to the SAW and allows for higher modulation rates. At room temperature, we observe an excitonic dynamics which is very sensitive to local laser-induced photodoping. At high laser excitation powers, instead of a decrease in PL intensity due to exciton dissociation by the traveling SAW field, we observe an unusual increase in some situations. The enhancement in PL emission is associated with the fact that, at higher acoustic powers, the SAW piezoelectric field partially reverses the photodoping which builds up prior to the application of the SAW. Our results unveil some of the main mechanisms underlying the interaction of different excitonic complexes with SAWs in vdWHs.

Figure 1(a) illustrates the experiment. The hBN/1L-WSe_2_/hBN vdWH was placed in front of a floating electrode unidirectional transducer (FEUDT) which controlled the SAW generation while the microphotoluminescence (μ-PL) measurements were performed. Figure 1(b) shows a typical 5 K μ-PL spectrum of the wdWH (black empty circles). The spectrum of 1L-WSe_2_ at low temperatures is composed of different excitonic contributions.^25−31^ The best fit to the spectrum (olive solid line) reproduces well the acquired data. The individual excitonic contributions are shown by the colored lines. The well-separated and highest energy component (blue) around 1.725 eV is attributed to X^0^.^30,32^ The orange and brown components are attributed to the recombination of XX and XX^–^,^28,30,32−34^ respectively, while the green and red components to the X_*intra*_^–^ and X_*inter*_^–^ recombinations,^32^ respectively. The purple component is possibly associated with dark excitons.^32,35−38^ In the Supporting Information, we detail the attribution of these excitonic lines. The emissions detected below 1.67 eV are position-dependent across the 1L-WSe_2_ region. They also respond to the acoustic modulation (see the Supporting Information).

**Figure 1:**
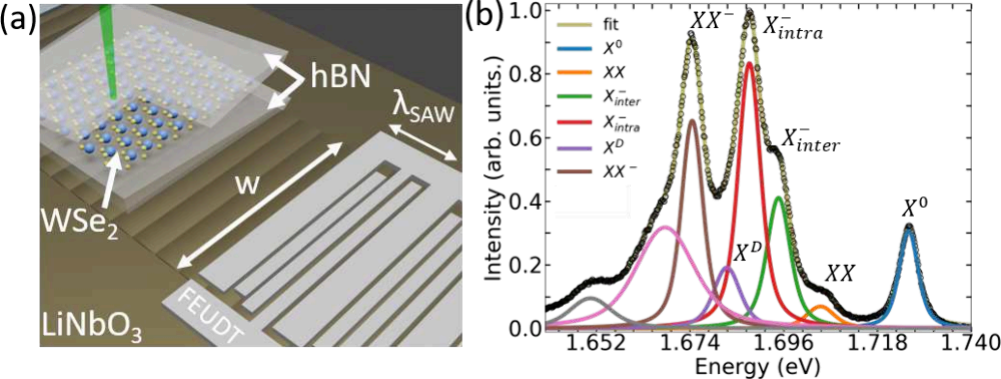
(a) Schematics of the experiment: the hBN/WSe_2_/hBN vdWH is placed in front a FEUDT device and optical excitation is performed using a green laser. (b) Normalized μ-PL spectrum of the sample at 5 K highlighting the contributions from neutral excitons (X^0^), intra (X_*intra*_^–^) and intervalley (X_*inter*_^–^) trions, dark excitons (X^D^), biexciton (XX) and negatively charged (XX^–^) biexcitons. The olive solid line is the fit to the data.

Figure 2(a) shows the (normalized) spectrally integrated μ-PL response of the vdWH at 5 K when the SAW is turned on and off as a function of time. During the measurement, the acoustic power is increased from 0 to 25 dBm. Between every PL measurement with the SAW on, three are performed with the SAW off. For powers above 1 dBm, when the SAW is turned on the PL emission is quenched and the quenching degree increases steadily up to approximately 50% at larger SAW powers. The PL intensity decrease at the laser generation spot is a consequence of the interaction between the SAW and the optically generated excitons. The spatially modulated SAW piezoelectric potential induces a type-II modulation, which laterally separates electrons and holes, increasing carrier lifetimes and enhancing nonradiative recombination rates. Part of the carriers is also captured in the maxima and minima of the traveling piezoelectric potential and dragged away from the laser generation spot, thus also contributing to the PL quenching.^39^ The SAW strain can also induce PL quenching via bandgap modulation.^40^ However, as we discuss in the Supporting Information, our experimental evidence indicates that the main contribution to the exciton dissociation in our vdWH comes from the SAW in-plane piezoelectric field.^17^ Another contribution to the PL quenching can come from impact ionization. Since carrier mobilities tend to be larger in vdWHs, the SAW piezoelectric field can accelerate carriers which transfer their energy to the excitons, ionizing them.^41^ This exciton dissociation mechanism depends on carrier mobility and, as we discuss below, can play a role in the temperature dependence of the X^0^ acoustic modulation.

**Figure 2:**
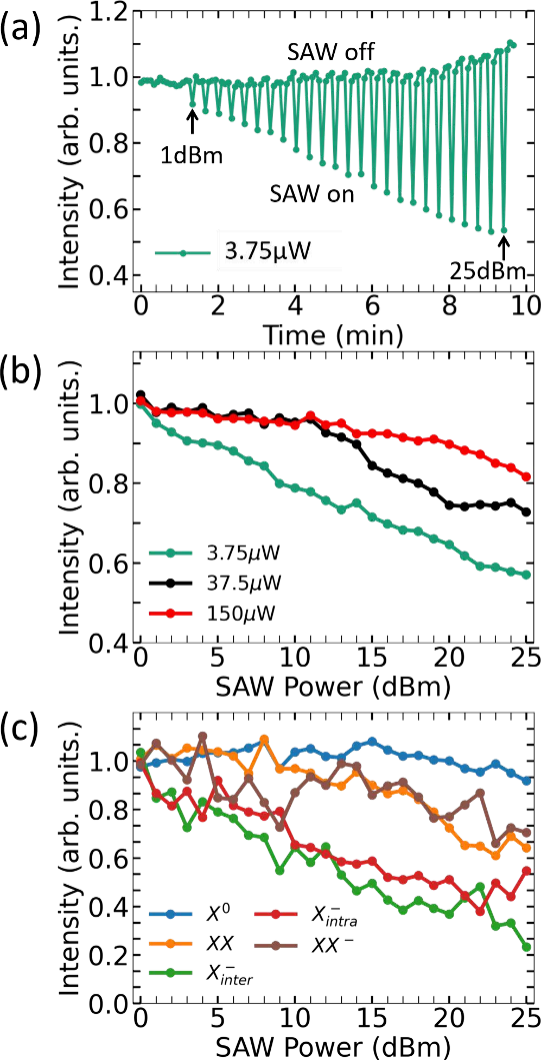
Acoustic modulation of the hBN/WSe_2_/hBN vdWH at 5 K. (a) Normalized and spectrally integrated μ-PL intensity as a function of time as the SAW power is turned on and off from 0 to 25 dBm in steps of 1 dBm. Laser excitation power is 3.75 μW. (b) Spectrally integrated and normalized μ-PL intensity as a function of the SAW power for different laser excitation powers. (c) Normalized μ-PL intensity (measured at 3.75 μW laser power) as a function of the SAW power for the different excitonic lines detected in the vdWH spectrum.

The measurements with the SAW on and off show that the excitonic response to the SAW stimulus is very fast and the PL intensity is immediately recovered after the SAW is turned off. This behavior is considerably different from what has been observed in pristine 1L-MoSe_2_ and 1L-MoS_2_ deposited directly on LiNbO_3_,^14,17,42^ demonstrating that the hBN encapsulation improves the 1L-WSe_2_ carrier mobility.^43^ It also confirms that, besides the 20 nm-thick hBN between the LiNbO_3_ and the 1L-WSe_2_, the SAW evanescent piezoelectric field is still large enough to efficiently interact with excitons and carriers in the TMD layer.

Figure 2(b) shows the spectrally integrated μ-PL response of the system as a function of the SAW power for different laser powers (for clarity, we omitted the measurements with the SAW off shown in Figure 2(a)). The PL quenching degree decreases consistently as the laser power is increased from 3.75 μW to 150 μW (for other laser power measurements see the Supporting Information). The reduction of the PL quenching degree at high laser powers has been reported for 1L-WSe_2_ at room temperature^16,19^ and is attributed to the screening of the SAW piezoelectric field at high carrier densities. Figure 2(c) shows the response of each individual excitonic emission at 5 K using the lowest laser excitation power, where such electrostatic effects are minimal. The least affected quasiparticle by the SAW piezoelectric field is X^0^. Its emission is roughly unaltered. The main reason for such weak interaction with the acoustic fields is the larger X^0^ binding energy (≈350 meV) as compared to the remaining excitonic complexes (between 21 and 53 meV), as presented in the Supporting Information. While placing the 1L-WSe_2_ (or any other 1L-TMD) directly on top of the LiNbO_3_ surface decreases the X^0^ binding energy due to the strong dielectric screening,^16,17^ the presence of the hBN layer in the vdWH restores the weak dielectric screening regime, thus increasing the X^0^ binding energy.^44−46^ As we discuss below, the lower mobilities at low temperatures also contribute for such weak response of X^0^ to the SAW modulation.

Charged exciton complexes like X_*intra*_^–^ and X_*inter*_^–^ can be easier dissociated due to their charged character and lower binding energies, which are much less sensitive to the dielectric environment as compared to the X^0^ binding energy.^47^Figure 2(c) shows that X_*intra*_^–^ and X_*inter*_^–^ are the most sensitive excitonic complexes to the SAW piezoelectric field. The XX and XX^–^ biexciton complexes also show a fast response to the SAW stimulus, but their quenching degree is intermediate as compared to X_*intra*_^–^ and X_*inter*_^–^. For the case of XX, despite its charge neutrality, it has the lowest binding energy the excitonic complexes discussed here. The XX^–^ response to the SAW is similar to XX. Although the uncertainty in the determination XX^–^ intensity is larger because of the low laser power employed in this experiment (Supporting Information), the similarity between the behavior of XX and XX^–^ can be related to the fact that XX^–^ owns an extra charge, but has the largest binding energy among the excitonic complexes. To our knowledge, the results presented in Figure 2(c) are the first demonstration of efficient interaction of both types of trions (X_*intra*_^–^ and X_*inter*_^–^) as well as biexciton complexes (XX and XX^–^) in TMD systems with SAWs. We also observed that, under nonresonant optical excitation, the SAW momentum does not affect the spin-valley scattering mechanisms in these excitonic complexes (Supporting Information).

We now address the exciton modulation at higher temperatures in the vdWH, when the biexciton emission vanishes. From here on, the X_*intra*_^–^ and X_*inter*_^–^ trions will be referred to as X^–^ due to the difficulty to distinguish the two components in the spectrum. Figure 3(a) presents color maps of the μ-PL intensity emitted by X^0^ at 5 (top), 100 (center) and 200 K (bottom panel) as a function of the laser and SAW powers. In all panels, the X^0^ μ-PL is normalized with respect to the emission measured without the SAW. Completing the information presented in Figure 2(c), at 5 K the X^0^ emission is weakly affected by the SAW. Its PL quenching degree is independent of the optical power (Figure 3(b)), thus showing that the screening of the excitonic emission with laser power (Figure 2(b)) affects primarily the higher order excitonic complexes like trions and biexcitons in the vdWH which have much lower binding energies as compared to X^0^. Moreover, these results indicate that at low temperatures, despite an overall improvement in mobility as compared to pristine monolayers, neutral excitons still own a localized character in the vdWH associated with potential fluctuations and disorder, as also observed recently in acoustic transport of interlayer excitons.^20^

**Figure 3:**
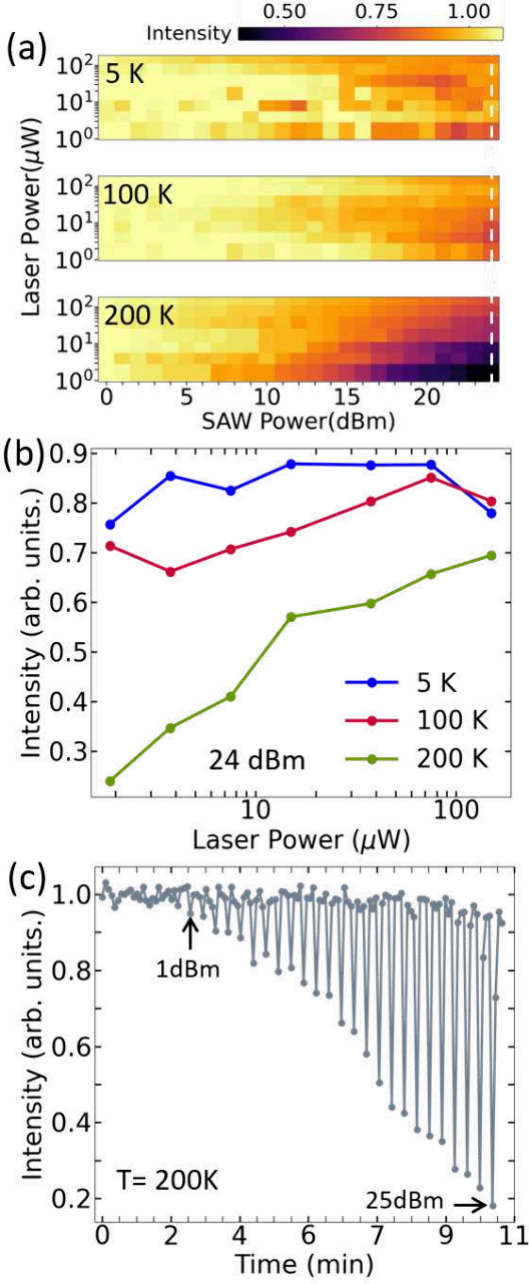
(a) Color maps showing the normalized X^0^ μ-PL intensity at 5, 100 and 200 K. The vertical axis shows the logarithm of the laser excitation power while the horizontal one brings the applied SAW power. (b) Profiles corresponding to the dashed white lines in (a) extracted for the X^0^ emission at 24 dBm. (c) Normalized μ-PL intensity as a function of time as the SAW power is turned on and off from 0 to 25 dBm measured at 1.87 μW and 200 K.

The 100 K color map in Figure 3(a) is slightly different. In this case, some PL quenching at higher acoustic powers and the effect of the laser power screening in the X^0^ emission are observed. This is evidenced in Figure 3(b), where the emission profiles measured at 24 dBm (white dashed lines in Figure 3(a)) are plotted. The PL quenching degree at low laser powers is larger at 100 K than at 5K, but they become very similar as the laser power is increased. This is more dramatic at 200 K, where much stronger PL quenching degrees of the X^0^ emission at low laser powers are measured and a diminished effect of the laser screening at high laser powers is observed. Figure 3(c) illustrates this strong interaction of the SAWs and X^0^ at 200 K by presenting the same kind of plot shown in Figure 2(a). Again, we see a very fast response of the PL emission as the SAW is turned on and off, but the quenching degree is considerably larger, reaching more than 80%. Above 130 K, the X^0^ population starts to be thermally dissociated^48,49^ (Supporting Information) which increases the number of free carriers in the system, making more relevant the role of impact ionization in driving the dissociation of X^0^ and the enhancement of its modulation with temperature. Exciton diffusion can also increase with temperature in the presence of disorder,^50,51^ further contributing to the rise in the degree of PL quenching of X^0^.

Figure 4(a) shows the spectrally integrated μ-PL of the vdWH at room temperature as a function of the SAW power. For the 1.5 μW laser excitation, as the SAW power increases the PL quenches as observed at low temperatures. However, for larger laser powers the scenario is very different. As the SAW power is increased, instead of quenching, there are situations where the PL intensity is enhanced. For some of the larger laser powers, the PL intensity starts to decrease and in the middle of the measurement it increases (highlighted by the gray dashed ellipses), as in the case of the 37.5 and 75 μW measurements. The sequence of measurements shown Figure 4(a) has been repeated on many sample locations and the results are very similar (Supporting Information). We did not identify any threshold in acoustic power or laser intensity which triggers such a PL behavior. However, statistically, the PL increase tends to appear at higher laser powers. Moreover, this unusual PL enhancement is observed only at room temperature, indicating that they are likely associated with metastable (carrier trap) states.

**Figure 4:**
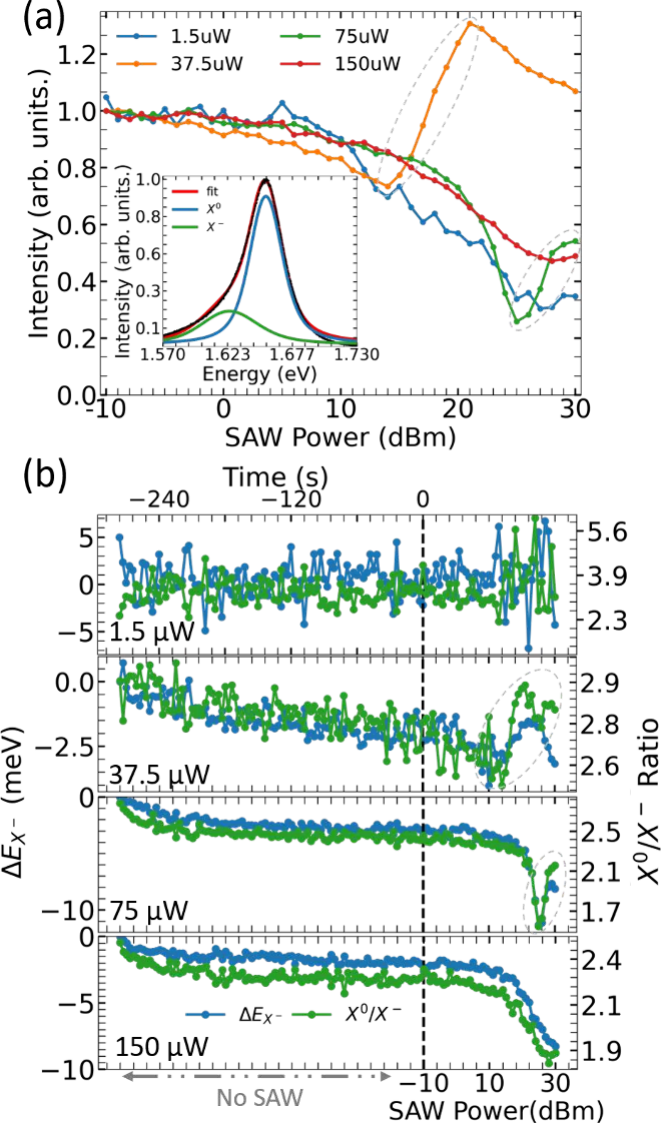
Acoustic modulation of the hBN/WSe_2_/hBN vdWH at room temperature. (a) Normalized and spectrally integrated μ-PL intensity for different laser excitation powers as a function of the SAW power. The inset shows the contributions from the X^0^ and X^–^ emissions to the spectrum of the vdWH without the SAW. (b) X^–^ energy shift (Δ*E*_X^–^_, left vertical scale, blue dots) and X^0^/X^–^ intensity ratio (green dots, right vertical scale) for the different laser excitation powers presented in (a) as a function of the SAW power. The behavior of Δ*E*_X^–^_ and the X^0^/X^–^ intensity ratio before the SAW is turned on is also shown. The dashed vertical line indicates when the SAW is turned on.

To understand the origin of such effects, Figure 4(b) presents the X^–^ emission energy shift (Δ*E*_X^–^_, left vertical scale) and the ratio between the X^0^ and X^–^ intensities (right vertical scale) for the laser powers shown in Figure 4(a). These two quantities started to be monitored before the SAW was applied (indicated by the negative time scale shown in the upper horizontal axis). The vertical dashed line indicates when the SAW is turned on. For 1.5 μW, neither Δ*E*_X^–^_ or the X^0^/X^–^ ratio are affected by the SAW. The absence of an acoustically induced Stark shift at low laser excitation powers has been consistently observed in all sample locations at room temperature. This is in agreement with the low in-plane polarizabilities expected for excitons in a weak dielectric screening regime induced by the sandwiching of the 1L-WSe_2_ with hBN.^52,53^ For a quantitative analysis of the expected Stark shift induced by the in-plane SAW piezoelectric field see the Supporting Information.

Before the SAW is turned on, for the high laser powers, both Δ*E*_X^–^_ and the X^0^/X^–^ ratio systematically change in time (and stabilize) in Figure 4(b): the X^–^ emission redshifts and the X^0^/X^–^ ratio reduces. The integrated μ-PL also decreases during this time (Supporting Information). Such an intensity decrease under continuous laser excitation is associated with laser-induced doping and exchange of carriers between the vdWH and its surroundings.^54,55^ Upon illumination, electrons can be injected into the 1L-WSe_2_ from defects states in hBN^56^ as well as from the substrate due to the small thickness of our bottom hBN layer,^57^ which is consistent with the decrease in the X^0^/X^–^ ratio prior to the application of the SAW. Therefore, the laser initially excites electrons and holes in the vdWH (depicted in Figure 5(a)). After a certain time under laser incidence, extra electrons are injected and diffuse across the vdWH (Figure 5(b)), leading to slow decrease in the X^0^/X^–^ ratio and PL redshift. Here, we exclude the influence of Auger effects in the X^0^/X^–^ ratio changes because of the time scale of events observed in Figure 4(b) and due to the strong suppression of this recombination mechanism in hBN-encapsulated systems.^58,59^

**Figure 5:**
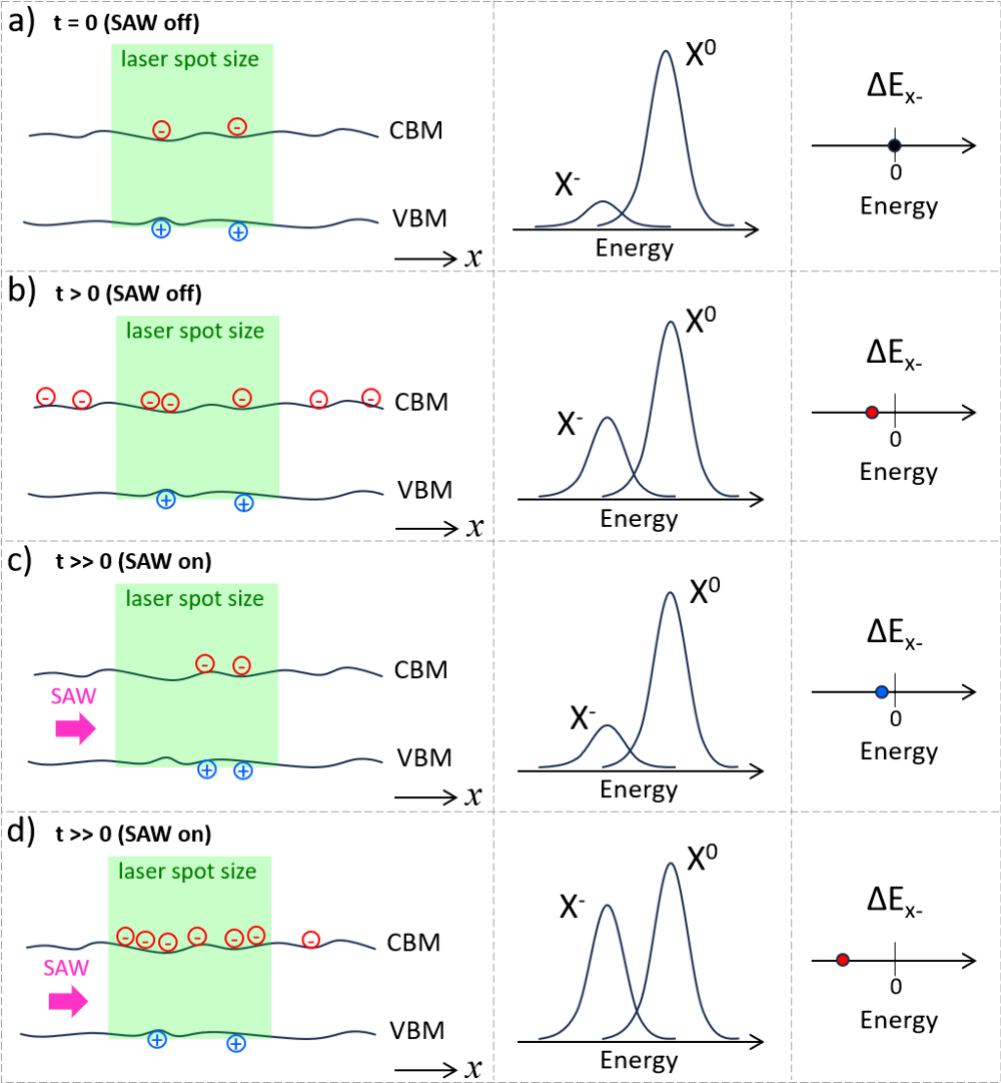
Charge and exciton dynamics at room temperature. (a) Initially (*t* = 0), the laser excites electrons and holes, leading to the X^0^ and X^–^ emissions (middle panel). (b) After some time of laser exposure (*t* > 0) the vdWH becomes electron-doped, increasing the X^–^ contribution to the optical emission and redshifting its emission (Δ*E*_X^–^_, right panel). (c) When the SAW is turned on, it can drag carriers away from the laser spot, partially reverting the laser-photodoping and blueshifting the X^–^ emission. (d) Conversely, the SAW can also drag electrons from trapped states back under the laser spot, further increasing the X^–^ contribution and redshifting its emission. In the left panels, *x* indicates the in-plane SAW propagation direction. CBM stands for conduction band minimum and VBM for valence band minimum profiles along *x*.

When the SAW is turned on, for 37.5 and 75 μW, Figure 4(b) shows that the PL enhancement observed in Figure 4(a) is followed by a strong increase in the X^0^/X^–^ ratio and a blueshift of the X^–^ emission (dashed ellipses). For 150 μW, even though the integrated PL steadily decreases as the SAW power is raised (Figure 4(a)), it continuously redshifts and the X^0^/X^–^ ratio only decreases. This is the opposite of what has been observed at 5 K (Figure 2(c), where the X^0^/X^–^ ratio increases because X^–^ is preferentially dissociated in comparison X^0^) and is another indication of electron injection in the system. Since no energy shifts associated with the SAW strain and piezoelectric field have been observed at 1.5 μW, the red and blueshifts detected at high laser excitation should have a purely electrostatic nature. We argue that such unusual PL behavior with SAW at high laser powers at room temperature is strongly linked to the laser-induced photodoping state reached by the structure prior to the application of the SAW and to the existence of a metastable carrier dynamics associated with the presence of saturated trapping centers. In this scenario, the SAW performs a dual role at room temperature, sometimes partially reverting the photodoping (PL blueshift) and sometimes rearranging and releasing trapped carriers (PL redshift).

The first role is clearly observed in the 37.5 μW and 75 μW measurements in Figure 4(b). When the acoustic power is increased, even though the SAW piezoelectric field might be partially screened by the photodoping, some of the carriers (mostly electrons) can be washed away from the laser excitation spot. When the SAW power is sufficiently high, the photodoping is partially reversed, as indicated by a recovery of the PL intensity (ellipses in Figure 4(a)) and a blueshift of the X^–^ emission (ellipses in Figure 4(b)), as depicted in Figure 5(c). The second role happens due to the carrier trapping and release process from localization centers around the laser spot. This is particularly important to understand the continuous redshift in the 150 μW measurement where, before the SAW is turned on, Δ*E*_X^–^_ is stabilized. Here, the SAW propagating field steadily releases carriers (which have been accumulated over time) from saturated trapping centers (like potential fluctuations or unintentional and undetectable residue from the sample fabrication). The release of these carriers leads to a further increase in the electron density, demonstrated by the X^0^/X^–^ ratio reduction and PL redshift as the SAW power is increased (Figure 5(d)). In other words, the SAW also seems to contribute to local sample doping by rearranging the carrier distribution on the 1L-WSe_2_ when the laser powers are relatively high. The time scale of the effects observed here are also consistent with persistent photoconductivity mechanisms induced by trapping of carriers in potential fluctuations due to sample inhomogeneity.^42,60,61^ The role of metastable localization states in the SAW-induced carrier dynamics at room temperature is important to understand the dependence of effect on the sample location and its absence at lower temperatures.

We probed the acoustically induced dynamics of optically generated excitons in hBN/1L-WSe_2_/hBN heterostructures. We showed how the different excitonic complexes detected at low temperature respond to a 250 MHz SAW propagating field. Inter and intravalley trions interact strongly with the SAW due to the combination of lower binding energy and extra charge. Biexcitons interact weaker with the SAW piezoelectric field as compared to trions due to their charge neutrality. Charged biexcitons have a similar response because, besides their extra charge, they have a considerably larger binding energy as compared to neutral biexcitons. As the temperature is increased, carrier localization effects are less effective, leading to a stronger interaction of neutral excitons with the SAW. At room temperature, the SAW-induced exciton dynamics is strongly affected by the presence of local laser-induced doping and metastable charge states. Our results contribute significantly to the understanding of excitonic dynamics under the influence high-frequency electric fields which is extremely valuable for applications of 2D materials and van der Waals heterostructures.

## Supporting Information Available

Experimental methods; photoluminescence characterization of the sample without the SAW; localized state emission modulation with the SAW; room temperature photoluminescence measurements with the SAW; circular polarization measurements with the SAW; methodology for curve fitting; and finite element simulations (PDF)

## References

[ref1] Nie X.; Wu X.; Wang Y.; Ban S.; Lei Z.; Yi J.; Liu Y.; Liu Y. Surface acoustic wave induced phenomena in two-dimensional materials. Nanoscale Horiz. 2023, 8, 158 10.1039/D2NH00458E. 36448884

[ref2] Zhao P.; Sharma C. H.; Liang R.; Glasenapp C.; Mourokh L.; Kovalev V. M.; Huber P.; Prada M.; Tiemann L.; Blick R. H. Acoustically Induced Giant Synthetic Hall Voltages in Graphene. Phys. Rev. Lett. 2022, 128, 256601 10.1103/PhysRevLett.128.256601. 35802443

[ref3] Fandan R.; Pedrós J.; Hernández-Mínguez A.; Iikawa F.; Santos P. V.; Boscá A.; Calle F. Dynamic Local Strain in Graphene Generated by Surface Acoustic Waves. Nano Lett. 2020, 20, 402 10.1021/acs.nanolett.9b04085. 31790600

[ref4] Zhao Q.; Yan H.; Wang X.; Chen Y.; Zhang S.; Wu S.; Huang X.; Di Y.; Xiong K.; Zeng J.; Jiao H.; Lin T.; He H.; Ge J.; Meng X.; Shen H.; Chu J.; Wang J. Significant Suppression of Dark Current in a Surface Acoustic Wave Assisted MoS_2_ Photodetector. Adv. Electron. Mater. 2023, 9, 2300496 10.1002/aelm.202300496.

[ref5] Alijani H.; Reineck P.; Komljenovic R.; Russo S. P.; Low M. X.; Balendhran S.; Crozier K. B.; Walia S.; Nash G. R.; Yeo L. Y.; Rezk A. R. The acoustophotoelectric effect: efficient Phonon-Photon-Electron Coupling in Zero-Voltage-Biased 2D SnS_2_ for Broad-Band Photodetection. ACS Nano 2023, 17, 19254 10.1021/acsnano.3c06075. 37755696

[ref6] Zheng S.; Wu E.; Feng Z.; Zhang R.; Xie Y.; Yu Y.; Zhang R.; Li Q.; Liu J.; Pang W.; Zhang H.; Zhang D. Acoustically enhanced photodetection by a black phosphorus–MoS_2_ van der Waals heterojunction p–n diode. Nanoscale 2018, 10, 10148 10.1039/C8NR02022A. 29785445

[ref7] Iikawa F.; Hernández-Mínguez A.; Aharonovich I.; Nakhaie S.; Liou Y.; Lopes J. M. J.; Santos P. V. Acoustically modulated optical emission of hexagonal boron nitride layers. Appl. Phys. Lett. 2019, 114, 1711047 10.1063/1.5093299.

[ref8] Zhang J.; Wu C.; Zhang Q.; Liu J. Mechano/acousto-electric coupling between ReS_2_ and surface acoustic wave. Nanotechnol. 2023, 34, 155501 10.1088/1361-6528/acb447. 36652706

[ref9] Wang G.; Chernikov A.; Glazov M. M.; Heinz T. F.; Marie X.; Amand T.; Urbaszek B. Excitons in Atomically Thin Transition Metal Dichalcogenides. Rev. Mod. Phys. 2018, 90, 021001 10.1103/RevModPhys.90.021001.

[ref10] Gao X. G.; Li X. K.; Xin W.; Chen X. D.; Liu Z. B.; Tian J. G. Fabrication, optical properties, and applications of twisted two-dimensional materials. Nanophotonics 2020, 9, 1717 10.1515/nanoph-2020-0024.

[ref11] Li S.; Wei K.; Liu Q.; Tang Y.; Jiang T. Twistronics and moiré excitonic physics in van der Waals heterostructures. Front. Phys. 2024, 10.1007/s11467-023-1355-6.

[ref12] Liao W.; Huang Y.; Wang H.; Zhang H. Van der Waals heterostructures for optoelectronics: progress and prospects. Appl. Mater. Today 2019, 16, 435 10.1016/j.apmt.2019.07.004.

[ref13] Ma Q.; Ren G.; Xu K.; Ou J. Z. Tunable Optical Properties of 2D Materials and Their Applications. Adv. Optical Mater. 2021, 9, 2001313 10.1002/adom.202001313.

[ref14] Sheng L.; Tai G.; Yin Y.; Hou C.; Wu Z. Layer-Dependent Exciton Modulation Characteristics of 2D MoS_2_ Driven by Acoustic Waves. Adv. Opt. Mater. 2021, 9, 2001349 10.1002/adom.202001349.

[ref15] Rezk A. R.; Carey B.; Chrimes A. F.; Lau D. W. M.; Gibson B. C.; Zheng C.; Fuhrer M. S.; Yeo L. Y.; Kalantar-zadeh K. Acoustically-Driven Trion and Exciton Modulation in Piezoelectric Two-Dimensional MoS_2_. Nano Lett. 2016, 16, 849 10.1021/acs.nanolett.5b02826. 26729449

[ref16] Datta K.; Li Z.; Lyu Z.; Deotare P. B. Piezoelectric Modulation of Excitonic Properties in Monolayer WSe_2_ under Strong Dielectric Screening. ACS Nano 2021, 15, 12334 10.1021/acsnano.1c04269. 34181857

[ref17] Scolfaro D.; Finamor M.; Trinchão L.; Rosa B. L. T.; Chaves A.; Santos P. V.; Iikawa F.; Couto O. D. D. Jr. Acoustically Driven Stark Effect in Transition Metal Dichalcogenide Monolayers. ACS Nano 2021, 15, 15371 10.1021/acsnano.1c06854. 34450007

[ref18] Patel S. D.; Parto K.; Choquer M.; Lewis N.; Umezawa S.; Hellman L.; Polishchuk D.; Moody G. Surface Acoustic Wave Cavity Optomechanics with Atomically Thin h-BN and WSe_2_ Single-Photon Emitters. PRX Quantum 2024, 5, 010330 10.1103/PRXQuantum.5.010330.

[ref19] Datta K.; Lyu Z.; Li Z.; Taniguchi T.; Watanabe K.; Deotare P. B. Spatiotemporally controlled room-temperature exciton transport under dynamic strain. Nat. Photonics 2022, 16, 242 10.1038/s41566-021-00951-3.

[ref20] Peng R.; Ripin A.; Ye Y.; Zhu J.; Wu C.; Lee S.; Li H.; Taniguchi T.; Watanabe K.; Cao T.; Xu X.; Li M. Long-range transport of 2D excitons with acoustic waves. Nat. Commun. 2022, 13, 1334 10.1038/s41467-022-29042-9. 35289330 PMC8921513

[ref21] Bühler D. D.; Weiß M.; Crespo-Poveda A.; Nysten E. D. S.; Finley J. J.; Müller K.; Santos P. V.; de Lima M. M. Jr.; Krenner H. J. On-chip generation and dynamic piezo-optomechanical rotation of single photons. Nature Commun. 2022, 13, 6998 10.1038/s41467-022-34372-9. 36384915 PMC9668908

[ref22] Bienfait A.; Satzinger K. J.; Zhong Y. P.; Chang H.-S.; Chou M.-H.; Conner C. R.; Dumur; Grebel J.; Peairs G. A.; Povey R. G.; Cleland A. N. Phonon-mediated quantum state transfer and remote qubit entanglement. Science 2019, 364, 368 10.1126/science.aaw8415. 31023919

[ref23] Dumur; Satzinger K. J.; Peairs G. A.; Chou M.-H.; Bienfait A.; Chang H.-S.; Conner C. R.; Grebel J.; Povey R. G.; Zhong Y. P.; Cleland A. N. Quantum communication with itinerant surface acoustic wave phonons. npj Quantum Inf 2021, 7, 173 10.1038/s41534-021-00511-1.

[ref24] Zalalutdinov M. K.; Robinson J. T.; Fonseca J. J.; LaGasse S. W.; Pandey T.; Lindsay L. R.; Reinecke T. L.; Photiadis D. M.; Culbertson J. C.; Cress C. D.; Houston B. H. Acoustic cavities in 2D heterostructures. Nature Commun. 2021, 12, 3267 10.1038/s41467-021-23359-7. 34075055 PMC8169679

[ref25] You Y.; Zhang X. X.; Berkelbach T. C.; Hybertsen M. S.; Reichman D. R.; Heinz T. F. Observation of biexcitons in monolayer WS_2_. Nat. Phys. 2015, 11, 477 10.1038/nphys3324.

[ref26] He Y. M.; Clark G.; Schaibley J. R.; He Y.; Chen M. C.; Wei Y. J.; Ding X. D.; Zhang Q.; Yao W.; Xu X.; Lu C. Y.; Pan J. W. Single quantum emitters in monolayer semiconductors. Nat. Nanotechnol. 2015, 10, 497 10.1038/nnano.2015.75. 25938571

[ref27] Plechinger G.; Nagler P.; Arora A.; Schmidt R.; Chernikov A.; del Aguila A. G.; Christianen P. C. M.; Bratschitsch R.; Schuller C.; Korn T. Trion fine structure and coupled spin–valley dynamics in monolayer tungsten disulfide. Nat. Commun. 2016, 7, 12715 10.1038/ncomms12715. 27586517 PMC5025800

[ref28] Mostaani E.; Szyniszewski M.; Price C. H.; Maezono R.; Danovich M.; Hunt R. J.; Drummond N. D.; Fal'ko V. I. Diffusion quantum Monte Carlo study of excitonic complexes in two-dimensional transition-metal dichalcogenides. Phys. Rev. B 2017, 96, 075431 10.1103/PhysRevB.96.075431.

[ref29] Courtade E.; Semina M.; Manca M.; Glazov M. M.; Robert C.; Cadiz F.; Wang G.; Taniguchi T.; Watanabe K.; Pierre M.; Escoffier W.; Ivchenko E. L.; Renucci P.; Marie X.; Amand T.; Urbaszek B. Charged excitons in monolayer WSe_2_: Experiment and theory. Phys. Rev. B 2017, 96, 085302 10.1103/PhysRevB.96.085302.

[ref30] Li Z.; Wang T.; Lu Z.; Khatoniar M.; Lian Z.; Meng Y.; Blei M.; Taniguchi T.; Watanabe K.; McGill S. A.; Tongay S.; Menon V. M.; Smirnov D.; Shi S. F. Direct Observation of Gate-Tunable Dark Trions in Monolayer WSe_2_. Nano Lett. 2019, 19, 6886 10.1021/acs.nanolett.9b02132. 31487988

[ref31] He Y.-M.; Iff O.; Lundt N.; Baumann V.; Davanco M.; Srinivasan K.; Hoefling S.; Schneider C. Cascaded emission of single photons from the biexciton in monolayered WSe_2_. Nat.Comunn. 2016, 7, 13409 10.1038/ncomms13409. PMC5109589 27830703

[ref32] Barbone M.; Montblanch A. R.-P.; Kara D. M.; Palacios-Berraquero C.; Cadore A. R.; De Fazio D.; Pingault B.; Mostaani E.; Li H.; Chen B.; Watanabe K.; Taniguchi T.; Tongay S.; Wang G.; Ferrari A. C.; Atatüre M. Charge-tuneable biexciton complexes in monolayer WSe_2_. Nat. Commun. 2018, 9, 3721 10.1038/s41467-018-05632-4. 30213951 PMC6137137

[ref33] Li Z.; Wang T.; Lu Z.; Jin C.; Chen Y.; Meng Y.; Lian Z.; Taniguchi T.; Watanabe K.; Zhang S.; Smirnov D.; Shi S.-F. Revealing the biexciton and trion-exciton complexes in BN encapsulated WSe_2_. Nat. Commun. 2018, 9, 3719 10.1038/s41467-018-05863-5. 30213927 PMC6137082

[ref34] Liu E.; van Baren J.; Lu Z.; Altaiary M. M.; Taniguchi T.; Watanabe K.; Smirnov D.; Lui C. H. Gate Tunable Dark Trions in Monolayer WSe_2_. Phys. Rev. Lett. 2019, 123, 027401 10.1103/PhysRevLett.123.027401. 31386514

[ref35] Zhou Y.; Scuri G.; Wild D. S.; High A. A.; Dibos A.; Jauregui L. A.; Shu C.; De Greve K.; Pistunova K.; Joe A. Y.; Taniguchi T.; Watanabe K.; Kim P.; Lukin M. D.; Park H. Probing dark excitons in atomically thin semiconductors via near-field coupling to surface plasmon polaritons. Nat. Nanotechnol. 2017, 12, 856 10.1038/nnano.2017.106. 28650440

[ref36] Wang G.; Robert C.; Glazov M. M.; Cadiz F.; Courtade E.; Amand T.; Lagarde D.; Taniguchi T.; Watanabe K.; Urbaszek B.; Marie X. In-Plane Propagation of Light in Transition Metal Dichalcogenide Monolayers: Optical Selection Rules. Phys. Rev. Lett. 2017, 119, 047401 10.1103/PhysRevLett.119.047401. 29341750

[ref37] Park K. D.; Jiang T.; Clark G.; Xu X.; Raschke M. B. Radiative control of dark excitons at room temperature by nano-optical antenna-tip Purcell effect. Nat. Nanotechnol. 2018, 13, 59 10.1038/s41565-017-0003-0. 29158602

[ref38] Li Z.; Wang T.; Jin C.; Lu Z.; Lian Z.; Meng Y.; Blei M.; Gao S.; Taniguchi T.; Watanabe K.; Ren T.; Tongay S.; Yang L.; Smirnov D.; Cao T.; Shi S.-F. Emerging photoluminescence from the dark-exciton phonon replica in monolayer WSe_2_. Nat. Commun. 2019, 10, 2469 10.1038/s41467-019-10477-6. 31171789 PMC6554274

[ref39] Couto O. D. D. Jr.; Hey R.; Santos P. V. Spin dynamics in (110) GaAs quantum wells under surface acoustic waves. Phys. Rev. B 2008, 78, 153305 10.1103/PhysRevB.78.153305.

[ref40] Rudolph J.; Hey R.; Santos P. Long-Range Exciton Transport by Dynamic Strain Fields in a GaAs Quantum Well. Phys. Rev. Lett. 2007, 99, 047602 10.1103/PhysRevLett.99.047602. 17358707

[ref41] Santos P. V.; Ramsteiner M.; Jungnickel F. Spatially-resolved photoluminescence in GaAs surface acoustic wave structures. Appl. Phys. Lett. 1998, 72, 2099 10.1063/1.121288.

[ref42] Preciado E.; Schuelein F. J.; Nguyen A. E.; Barroso D.; Isarraraz M.; von Son G.; Lu I.-H.; Michailow W.; Moeller B.; Klee V.; Mann J.; Wixforth A.; Bartels L.; Krenner H. J. Scalable Fabrication of a Hybrid Field-Effect and Acousto-Electric Device by Direct Growth of Monolayer MoS_2_/LiNbO_3_. Nat. Commun. 2015, 6, 8593 10.1038/ncomms9593. 26493867 PMC4639816

[ref43] Cadiz F.; Courtade E.; Robert C.; Wang G.; Shen Y.; Cai H.; Taniguchi T.; Watanabe K.; Carrere H.; Lagarde D.; Manca M.; Amand T.; Renucci P.; Tongay S.; Marie X.; Urbaszek B. Excitonic Linewidth Approaching the Homogeneous Limit in MoS_2_-Based van der Waals Heterostructures. Phys. Rev. X 2017, 7, 021026 10.1103/PhysRevX.7.021026.

[ref44] Mak K. F.; Shan J. Photonics and Optoelectronics of 2D Semiconductor Transition Metal Dichalcogenides. Nat. Photonics 2016, 10, 216 10.1038/nphoton.2015.282.

[ref45] Raja A.; Chaves A.; Yu J.; Arefe G.; Hill H. M.; Rigosi A. F.; Berkelbach T. C.; Nagler P.; Schueller C.; Korn T.; Nuckolls C.; Hone J.; Brus L. E.; Heinz T. F.; Reichman D. R.; Chernikov A. Coulomb Engineering of the Bandgap and Excitons in Two-Dimensional Materials. Nature Commun. 2017, 8, 15251 10.1038/ncomms15251. 28469178 PMC5418602

[ref46] Goryca M.; Li J.; Stier A. V.; Taniguchi T.; Watanabe K.; Courtade E.; Shree S.; Robert C.; Urbaszek B.; Marie X.; Crooker S. A. Revealing Exciton Masses and Dielectric Properties of Monolayer Semiconductors with High Magnetic Fields. Nat. Commun. 2019, 10, 4172 10.1038/s41467-019-12180-y. 31519909 PMC6744484

[ref47] Van Tuan D.; Yang M.; Dery H. Coulomb Interaction in Monolayer Transition-Metal Dichalcogenides. Phys. Rev. B 2018, 98, 125308 10.1103/PhysRevB.98.125308.

[ref48] Huang J.; Hoang T. B.; Mikkelsen M. H. Probing the origin of excitonic states in monolayer WSe_2_. Sci. Rep. 2016, 6, 22414 10.1038/srep22414. 26940069 PMC4778068

[ref49] Zhang X.-X.; You Y.; Zhao S. Y. F.; Heinz T. F. Experimental Evidence for Dark Excitons in Monolayer WSe_2_. Phys. Rev. Lett. 2015, 115, 257403 10.1103/PhysRevLett.115.257403. 26722944

[ref50] Kato T.; Kaneko T. Transport Dynamics of Neutral Excitons and Trions in Monolayer WS_2_. ACS Nano 2016, 10, 9687 10.1021/acsnano.6b05580. 27666319

[ref51] Glazov M. M. Quantum Interference Effect on Exciton Transport in Monolayer Semiconductors. Phys. Rev. Lett. 2020, 124, 166802 10.1103/PhysRevLett.124.166802. 32383933

[ref52] Cavalcante L. S. R.; da Costa D. R.; Farias G. A.; Reichman D. R.; Chaves A. Stark Shift of Excitons and Trions in Two-Dimensional Materials. Phys. Rev. B 2018, 98, 245309 10.1103/PhysRevB.98.245309.

[ref53] Massicotte M.; Vialla F.; Schmidt P.; Lundeberg M. B.; Latini S.; Haastrup S.; Danovich M.; Davydovskaya D.; Watanabe K.; Taniguchi T.; Fal’ko V.; Thygesen K. S.; Pedersen T. G.; Koppens F. H. Dissociation of Two-Dimensional Excitons in Monolayer WSe_2_. Nat. Commun. 2018, 9, 1633 10.1038/s41467-018-03864-y. 29691376 PMC5915447

[ref54] Cadiz F.; Robert C.; Wang G.; Kong W.; Fan X.; Blei M.; Lagarde D.; Gay M.; Manca M.; Taniguchi T.; Watanabe K.; Amand T.; Marie X.; Renucci P.; Tongay S.; Urbaszek B. Ultra-Low Power Threshold for Laser Induced Changes in Optical Properties of 2D Molybdenum Dichalcogenides. 2D Materials 2016, 3, 045008 10.1088/2053-1583/3/4/045008.

[ref55] Orsi-Gordo V.; Balanta M. A. G.; Galvão Gobato Y.; Covre F. S.; Galeti H. V. A.; Iikawa F.; Couto O. D. D. Jr.; Qu F.; Henini M.; Hewak D. W.; Huang C. C. Revealing the nature of low-temperature photoluminescence peaks by laser treatment in van der Waals epitaxially grown WS_2_ monolayers. Nanoscale 2018, 10, 4807 10.1039/C8NR00719E. 29469923

[ref56] Aftab S.; Akhtar I.; Seo Y.; Eom J. WSe_2_ Homojunction p-n Diode Formed by Photoinduced Activation of Mid-Gap Defect States in Boron Nitride. ACS Appl. Mater. Interfaces 2020, 12, 42007 10.1021/acsami.0c12129. 32814429

[ref57] Jadczak J.; Glazov M.; Kutrowska-Girzycka J.; Schindler J. J.; Debus J.; Ho C.-H.; Watanabe K.; Taniguchi T.; Bayer M.; Bryja L. Upconversion of Light into Bright Intravalley Excitons via Dark Intervalley Excitons in hBN-Encapsulated WSe_2_ Monolayers. ACS Nano 2021, 15, 19165 10.1021/acsnano.1c08286. 34735768 PMC8717626

[ref58] Hoshi Y.; Kuroda T.; Okada M.; Moriya R.; Masubuchi S.; Watanabe K.; Taniguchi T.; Kitaura R.; Machida T. Suppression of exciton-exciton annihilation in tungsten disulfide monolayers encapsulated by hexagonal boron nitrides. Phys. Rev. B 2017, 95, 241403 10.1103/PhysRevB.95.241403.

[ref59] Zipfel J.; Kulig M.; Perea-Causín R.; Brem S.; Ziegler J. D.; Rosati R.; Taniguchi T.; Watanabe K.; Glazov M. M.; Malic E.; Chernikov A. Exciton diffusion in monolayer semiconductors with suppressed disorder. Phys. Rev. B 2020, 101, 115430 10.1103/PhysRevB.101.115430.

[ref60] Lee J.; Pak S.; Lee Y. W.; Cho Y.; Hong J.; Giraud P.; Shin H. S.; Morris S. M.; Sohn J. I.; Cha S.; Kim J. M. Monolayer Optical Memory Cells Based on Artificial Trap-Mediated Charge Storage and Release. Nature Commun. 2017, 8, 14734 10.1038/ncomms14734. 28337979 PMC5376667

[ref61] Pak J.; Lee I.; Cho K.; Kim J. K.; Jeong H.; Hwang W. T.; Ahn G. H.; Kang K.; Yu W. J.; Javey A.; Chung S.; Lee T. Intrinsic Optoelectronic Characteristics of MoS_2_ Phototransistors via a Fully Transparent van der Waals Heterostructure. ACS Nano 2019, 13, 9638 10.1021/acsnano.9b04829. 31345021

