# Supplementary material for: Acoustic Modulation of Excitonic Complexes in hBN/WSe2/hBN Heterostructures
Source: Nano Lett. 2024 Nov 25;24(49):15517–24. doi: 10.1021/acs.nanolett.4c03301 (PMC11638947; doi:10.1021/acs.nanolett.4c03301)
Supplement: Supplementary file 1 — nl4c03301_si_001.pdf [file nl4c03301_si_001.pdf]

Supporting Information
Acoustic modulation of excitonic complexes in hBN/WSe_2/hBN heterostructures

Marcos L. F. Gomes,[†] Pedro W. Matrone,[‡] Alisson R. Cadore,[¶] Paulo V. Santos,[§] and Odilon D. D. Couto Jr.[*,‖]

†*Universidade Estadual de Campinas, Instituto de Física Gleb Wataghin, 13083-859 Campinas, Brazil*

‡*Universidade Estadual de Campinas, Instituto de Física "Gleb Wataghin", 13083-859 Campinas, Brazil*

¶*Laboratório Nacional de Nanotecnologia, Centro Nacional de Pesquisa em Energia e Materiais, 13083-100 Campinas, Brazil*

§*Paul-Drude-Institut für Festkörperelektronik, Leibniz-Institut im Forschungsverbund Berlin e.V., Hausvogteiplatz 5-7, 10117 Berlin, Germany*

‖*Instituto de Física "Gleb Wataghin", Universidade Estadual de Campinas, 13083-859 Campinas, Brazil*

E-mail: odilon@ifi.unicamp.br

Experimental methods

Delay lines with FEUDTs (acoustic wavelength $\lambda_{SAW} = 28$ μm and width W = 424 μm) were lithographically fabricated on black Y-128° $LiNbO_3$ substrates. The vdWHs were fabricated following the procedure described in reference.[1] With WSe_2 and hBN crystals sourced from HQ Graphene, ultra thin flakes were obtained from mechanical exfoliation. The heterostructure composed by (≈10nm)hBN/1L-WSe2/(≈20nm)hBN onto $LiNbO_3$ substrate was fabricated by applying the well-established van der Walls pick-up technique using the policarbonate-based method.[1]

The optical spectroscopy measurements were performed in a cold finger He cryostat. Excitation was carried out using a continuous wave solid-state laser (532 nm) focused with a 50X magnification optical objective (2 μm spot size), also used to collect the μ-PL emission. The detection was performed with a 55 cm single-spectrometer (600 gr/mm gratings) and a CCD camera. The SAW experiments were performed at 250MHz (second harmonic of the FEUDT devices). Laser power, SAW generation and μ-PL detection were synchronized and controlled remotely.

Sample characterization without the SAW

Attribution of emission lines to excitonic complexes

Figure S1(a) shows the optical image of the vdWH. The blue (yellow) lines indicate the top (bottom) hBN layer while the green line limits the 1L-WSe_2 region. Figure S1(b) shows the 5 K μ-PL map (1μm step) of the 1L-WSe_2 region corresponding to the dashed-rectangular area highlighted in Figure S1(a). For clarity, Figure S1(c) reproduces the μ-PL spectrum measured at 5 K shown in Figure 1(b) of the main text (in a wider energy range), showing again the different individual excitonic contributions. The individual excitonic contributions

to the spectrum are shown by the colored lines. The laser power dependence for the six higher energy components is shown in Figure S1(d). The well-separated and highest energy component around 1.725 eV is attributed to the neutral exciton (X^0).[2,3] The orange and brown components which become stronger at high laser powers present power law exponents of 1.6 and 1.7, respectively, differing considerably from the linear behavior of the X^0 emission. The binding energies (with respect to X^0) extracted from Figure S1(c) for the orange and brown components are 21 and 53 meV, respectively. These are consistent with other literature experimental and theoretical reports, which assign them to the recombination of biexcitons (XX) and negatively charged biexcitons (XX^-).[2–6] The emissions denoted as $X^-{}_{intra}$ and $X^-{}_{inter}$ present a linear behavior with laser excitation (power law exponents of 1.1). Their binding energies are 31 and 38 meV and they are, therefore, assigned to the negatively-charged intravalley ($X^-{}_{intra}$) and negative-charged intervalley ($X^-{}_{inter}$) excitons, respectively.[2] The contribution observed 45 meV below the X^0 emission (purple line) is possibly related to dark excitons (X^D).[2,7,8] Its linear increase with laser power (power law exponent equal to 0.9) is also consistent with other observations.[9,10] Table 1 compares the binding energies found by fitting our spectra and those reported by M. Barbone *et.al.*[2]

The number and energy of the emission lines detected below 1.67 eV (here all labeled as X^{LS}) were observed to be position dependent across the 1L-WSe_2 region. As we see in Figure S1(c) (and Figure S7(a)), four LS components were usually necessary to fit the data. In some places, due to the lower intensities around 1.62 eV, three broader ones adjusted the spectra satisfactorily. This is why we focused our analysis on the main excitonic lines, since they are found consistently all over the WSe_2 region of the sample. However, as shown in Figure S7, the LS emissions also respond to the acoustic modulation with the SAW.

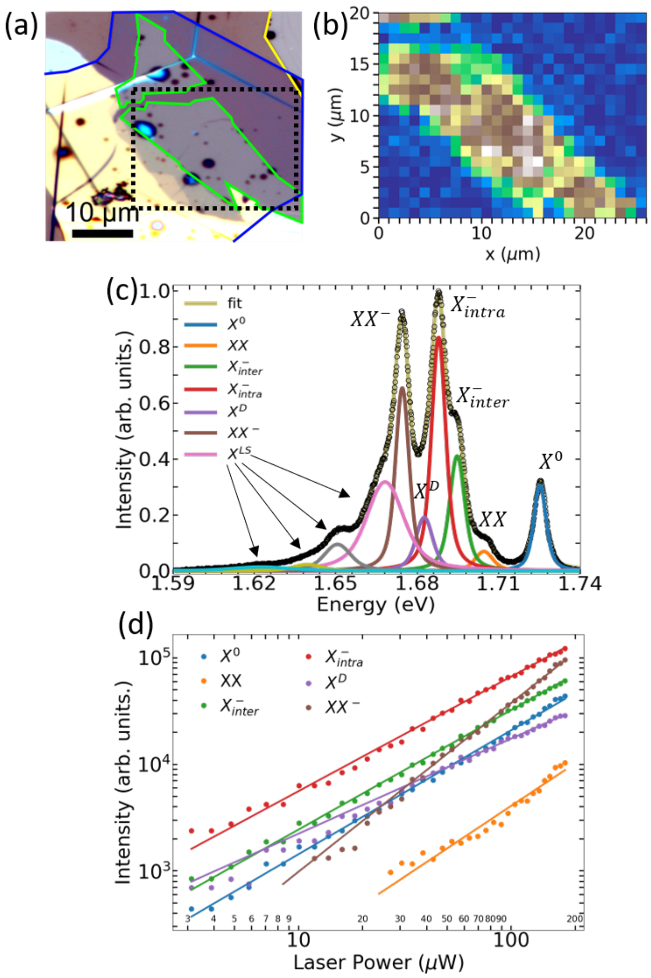

Figure S1: (a) hBN/WSe$_2$/hBN vdWH: colored lines indicate 1L-WSe$_2$ (green), upper (blue) and lower (yellow) hBN borders. The dashed square indicates the region where the measurement presented in (b) was performed. (b) μ-PL emission map of the structure at 5K without the SAW. (c) Reproduction of the spectrum shown in Figure 1(b) of the main text highlighting the different LS states also detected in the spectrum. (d) Laser excitation power dependence of the difference excitonic emissions of the sample at 5K corresponding to the spectrum shown in Figure 1(b) of the main text.

Table 1: Binding energies (E_B), with respect to the X^0 emission, for the different excitonic complexes identified in our hBN/ WSe_2/hBN heterostructures and those reported in Ref.[2]

Exciton	E_B (meV) (our measurements)	E_B (meV) from Ref.[2]
X^0	0	0
XX	21	17
$X^{-}{}_{intra}$	32	29
$X^{-}{}_{inter}$	39	36
X^D	45	43
XX^-	53	49

Temperature dependence of the X^0 emission

Figure S2 presents the X^0 PL intensity (without the SAW) as a function of the inverse temperature measured in our vdWH.

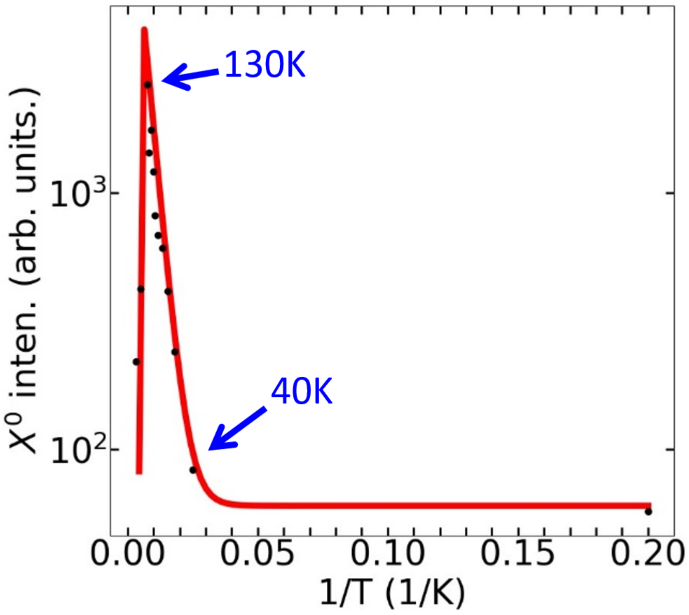

Figure S2: X^0 PL intensity (without the SAW) as a function of inverse temperature. The red curve is a fit with equation 1.

The red curve is a fit with the equation 1:[11]

$$I(T) = I(0)\frac{1 + Ae^{-\frac{E_1}{k_B T}}}{1 + Be^{-\frac{E_2}{k_B T}}} \quad (1)$$

where $I(0)$ is the PL intensity at $T = 0$ K, k_B the Boltzmann constant, and A and B are fitting parameters. E_1 is the activation energy associated to the increase in PL intensity with increasing temperature due to the presence of dark excitons. E_2 represents the activation energy for thermal exciton quenching process at higher temperatures.

From the fit with obtain E_1 = (27±4)meV and E_2 = (350±50)meV. E_1 corresponds to the energy splitting between dark and bright exciton states,[12] thus showing that the strong enhancement in PL emission above 40K is due to thermal population of bright exciton states from the lower energy dark ones. E_2 is similar to the expected value of the X^0 binding energy in WSe_2 on hBN, which is much larger compared to the values expected for WSe_2 directly on $LiNbO_3$.[13] We can also see that the onset of the thermal dissociation of neutral excitons is slightly above 130K. Therefore, the enhancement of X^0 acoustic modulation rates at 200K demonstrated in Figure 3(c) of the manuscript has a contribution from thermal effects. Thermal dissociation of the excitons enhances the number of free carriers in the system which contributes to the enhancement of dissociation of by impact ionization.

Low temperature measurements with SAWs

Fitting of the spectra for different SAW powers

As discussed in Figure S1(c), part of our analysis is based on multipeak fitting of the vdWH spectra under different experimental conditions. As mentioned before, at 5K six main excitonic components were always needed to fit the spectra above 1.67eV (a smaller number of components does not reproduce satisfactorily the experimental data). The energy position of these components (Table 1) and their laser power dependence (Figure S1(d)) are in very

good agreement with what has been observed so far for the spectrum 1L-WSe2 at 5K.[2–6]

The results presented in Figure 2(c) of the main text were obtained after fitting of the vdWH spectra measured for different SAW powers. Figure S3 summarizes this procedure by showing the spectra, the fit and corresponding peak components from 1 to 22dBm in steps of 3dBm. All the spectra were fitted with ten components (as in Figure S1(c)): six main excitonic lines and four LS components. As we see, the fit reproduces very well the measured spectra and allows us to track the behavior of each component as the SAW power is increased.

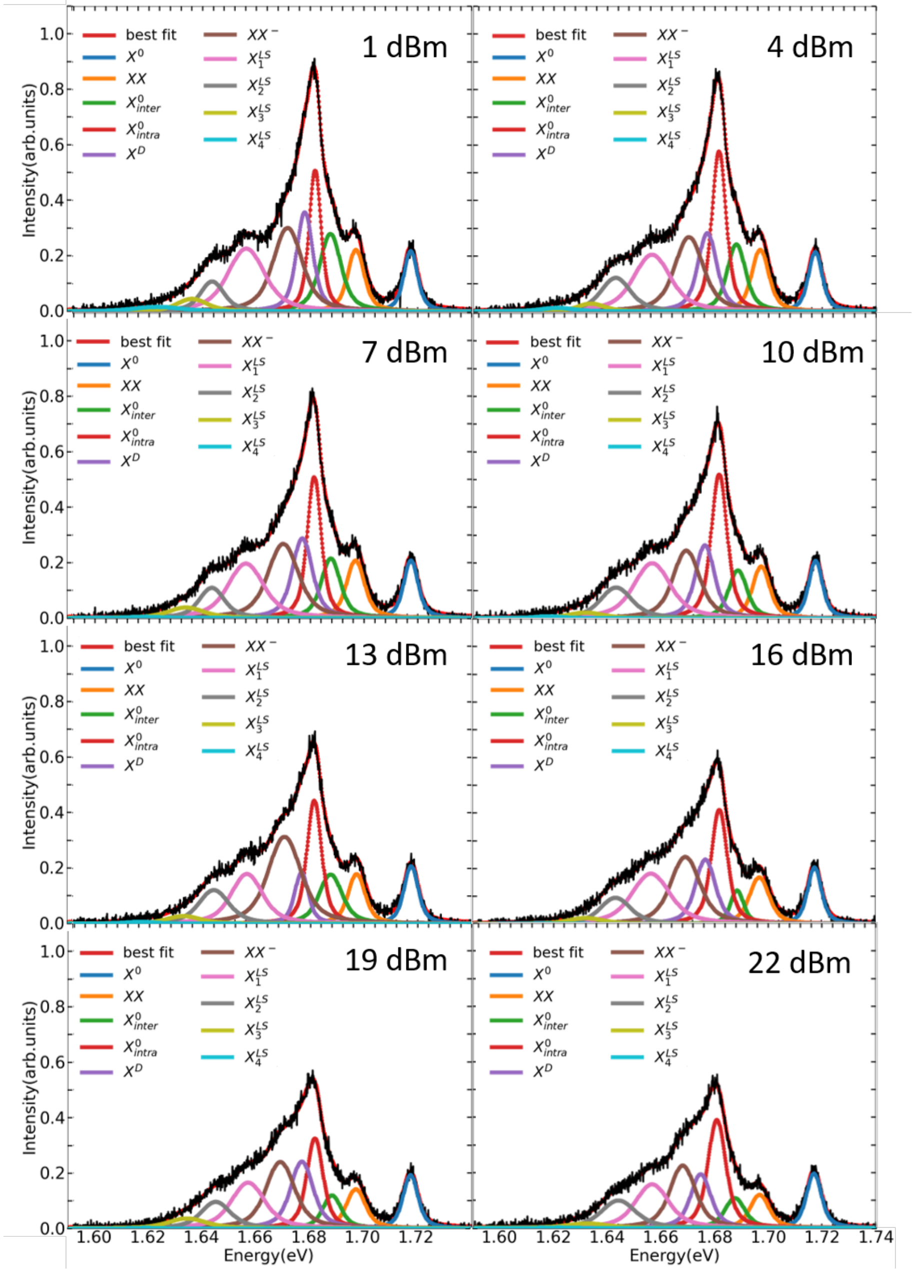

Figure S3: Spectra acquired at 5K as a function of the SAW power (every 3dBm) and their corresponding fit with the different spectral components: the main excitonic emissions (X^0, XX, X^-_{intra},X^-_{inter},X^D and XX^-) and the here called LS emissions (X^1_{LS}, X^2_{LS}, X^3_{LS}, X^4_{LS}). The spectra are normalized with respect to the spectra acquired before the SAW is turned on.

Figure S4 presents the data for each of the excitonic complexes shown in Figure 2(c) of the main text with error bars obtained from the fit procedure. As we can see, the error is larger for the XX^- emission, possibly due to the fact that, at such a low laser excitation

power, the charged biexciton emission has a similar intensity as compared to the spectrally close LS states.

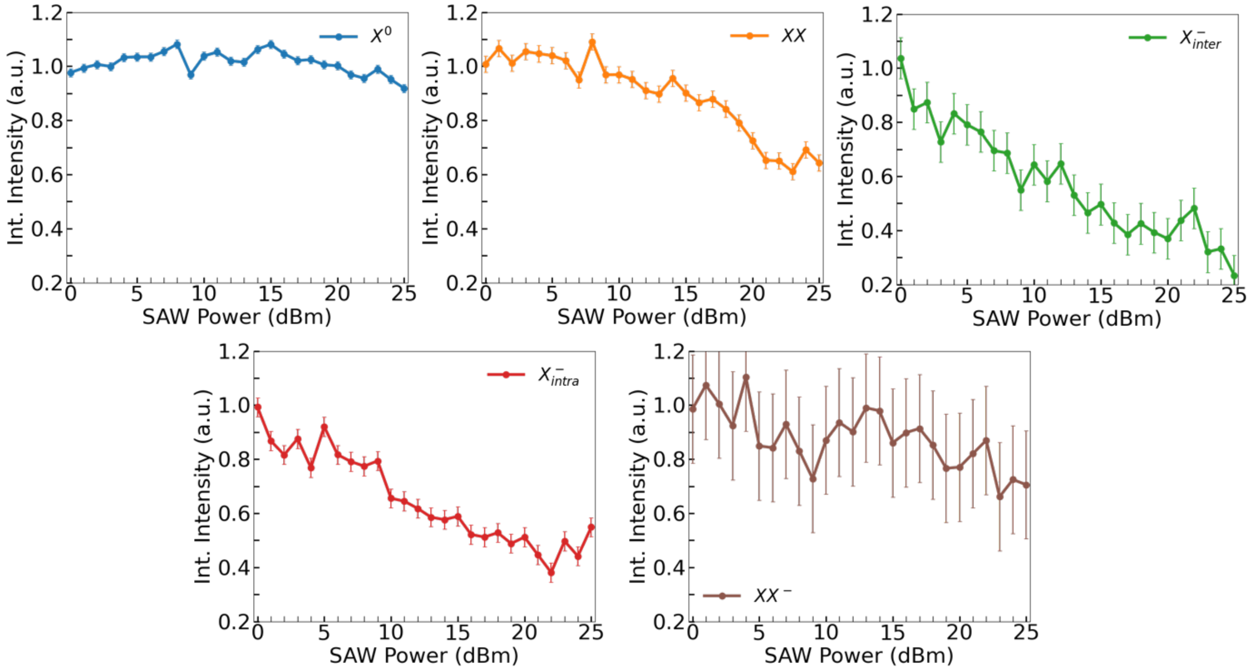

Figure S4: SAW power dependence of the intensity of the main excitonic complexes detected at 5K: X^0; XX; X^-_{intra}; X^-_{inter}; XX^-.

Circularly polarized PL detection

For circular polarization measurements, a broadband quarter wavelength plate was used to generate circularly polarized incidence on the samples and a polarization displacement prism was used to simultaneously detect the right (σ_+) and left (σ_-) circularly polarized PL components.

The degree of circularly polarized PL emitted from the samples is given by:

$$\rho = \frac{I^{\sigma+} - I^{\sigma-}}{I^{\sigma+} + I^{\sigma-}} \tag{2}$$

where $I^{\sigma+}$ and $I^{\sigma-}$ are the PL intensities for the right and left circular polarizations, respectively.

Figure S5 presents the measured degree o circular polarization of the main excitonic lines as a function of the SAW power. As we can see, neither the SAW strain or piezoelectric field are strong enough (at these acoustic powers) to increase the valley scattering mechanisms and change the degree of circularly polarized PL emission from the sample at non-resonant optical excitation conditions.

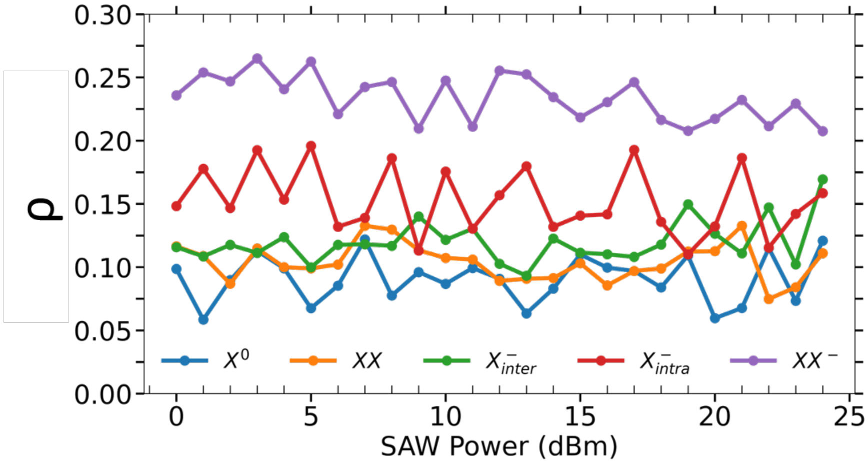

Figure S5: Degree of circularly polarized PL as a function of the SAW power measured at 5 K for the main excitonic emissions discussed in the main text.

Effect of laser excitation power

Figure S6 complements what has been shown in Figure 2(b) of the main text. There, for clarity, we chose to show only some of the laser power measurements which have been performed at 5K. Here, we present all the data.

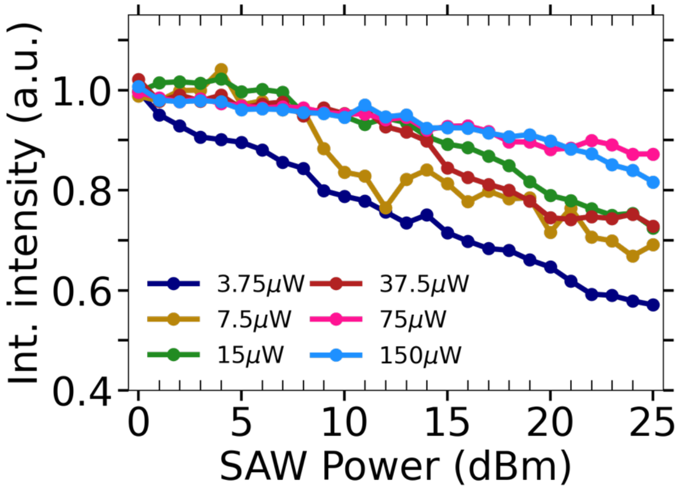

Figure S6: Spectrally integrated and normalized μ-PL intensity (at 5 K) as a function of the SAW power for all the different laser excitation powers.

SAW modulation of localized-state emission

Figure S7(a) shows a spectrum detected on the sample at 5 K with different emission lines below the XX^-. In this case, we observe four other components (labeled X_1^{LS}, X_2^{LS}, X_3^{LS} and X_4^{LS}) which possibly originate from strongly localized exciton emissions. Figure S7(b) shows spectrally integrated the response of these extra emissions at 3.75 μW laser power when the SAW is increased from 0 to 25dBm. As in the case of the main excitonic emissions discussed in the main text, these localized emission can also be turned on and off with a relatively fast rate using the SAW fields. Figure S7(c) depicts that they are also affected by the screening of the SAW piezoelectric at higher laser powers. Figure S7(d) shows the response of the individual four components which sum up to provide the data shown in Figure S7(b).

Room temperature measurements with SAWs

Figure 4(a) of the main text presents the behavior of the integrated PL emission at room temperature as a function of the applied SAW power which have been obtained under different

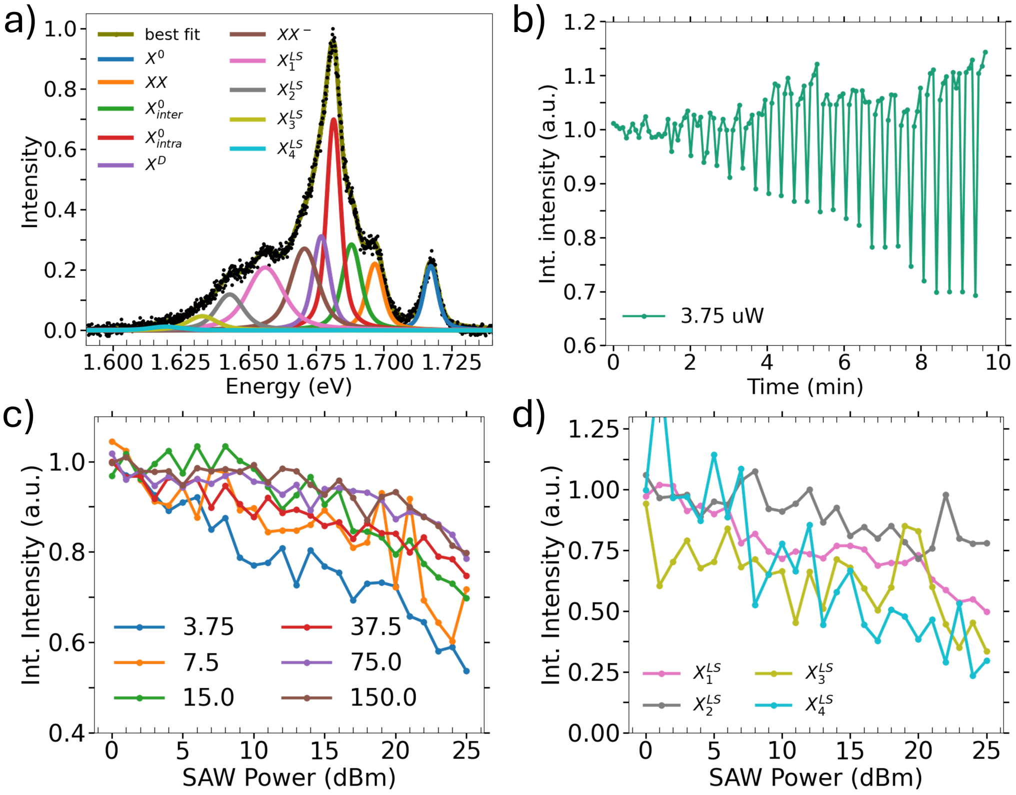

Figure S7: Effect of the SAW on the PL emission of lower (than the XX^-) energy states (LS). (a) Emission spectrum at 5 K. (b) Integrated (over the X_1^{LS}, X_2^{LS}, X_3^{LS} and X_4^{LS} states shown in (a)) PL as a function of time. (c) Integrated PL emission as a function of time for different laser excitation powers. (d) Acoustic modulation of the X_1^{LS}, X_2^{LS}, X_3^{LS} and X_4^{LS} states detected below the XX^- emission.

laser excitation conditions. There, we observe that in some situations, instead of quenching, the PL intensity increases as the SAW power is ramped up. In order to complement the information and better illustrate the result, in Figure S8 we present the raw spectra obtained for an acoustic power of 21 dBm. The blue spectra were acquired right before the SAW is turned on and the orange ones when the 21 dBm modulation is applied. We observe that, for the 1.5, 75 and 150 μW laser powers and 21dBm the PL emission decreases when the SAW is on, while for the 37.5 μW it increases, as presented in Figure 4(a).

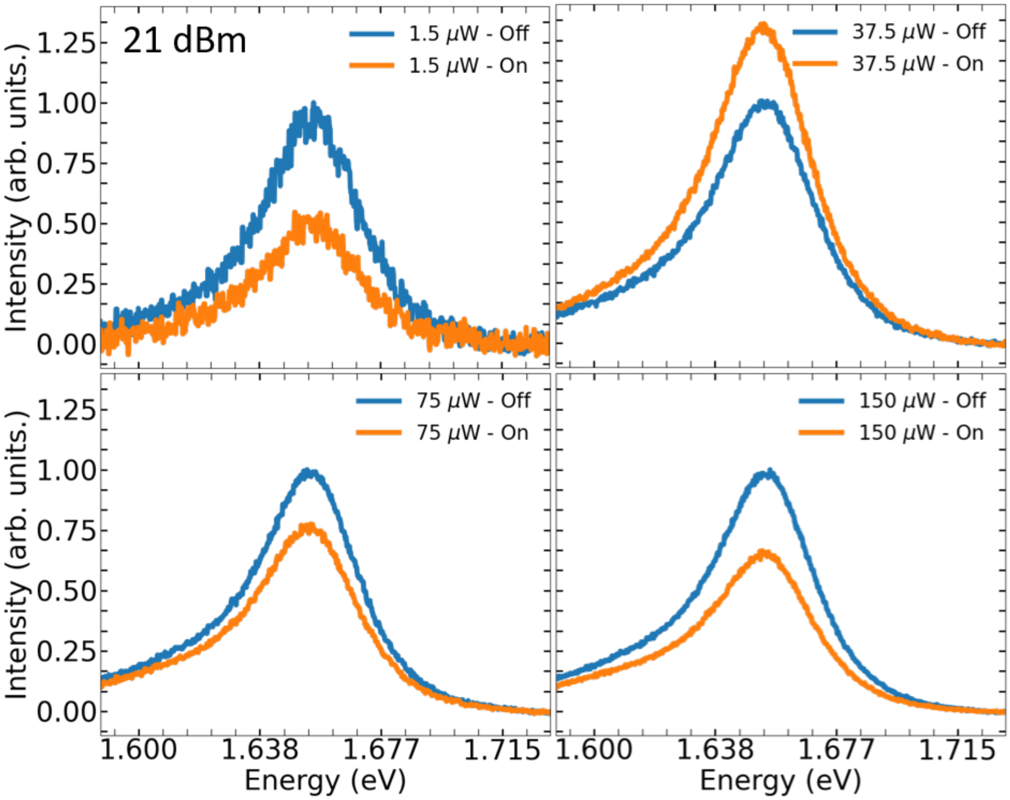

Figure S8: The plots show the spectra acquired with a SAW of 21dBm at 4 different laser excitation conditions at room temperature. Blue spectra are acquired with the SAW off and orange ones with the SAW on. The spectra were normalized with respect to the measurement performed with the SAW off.

Due to such unexpected behavior, all the room temperature measurements were performed in cycles consisting of 3 consecutive measurements where the SAW is increased from -10 to 30dBm in steps of 1dBm, as shown in Figure S9(a). The idea was to see if the PL increase observed when the SAW power is enhanced at room temperature could be due to any sort of instability in the μPL system (like mechanical) and if the behavior was reproducible (and not transitory). As we can see in Figure S9(a), the pattern of increase or quench in the PL is the very same in each cycle, independent of the laser power, thus showing that the result is reproducible and not transitory. It is also important to mention that in these measurements the laser power is remotely controlled with a motorized ND filter wheel, minimizing any mechanical instability during the change of laser excitation power. The viewgraph

in Figure S9(b) indicates 7 different points on the sample where these measurements were performed. Figure S9(c) presents the PL intensity (averaged over the 3 cycles) as a function of the SAW power for these 7 points under 3 different laser excitation powers. We observe that the PL intensity behavior is different for each point on the sample. This becomes clearer at high laser powers, where the increase (or decrease) in PL intensity happens at completely different SAW powers. This local dependence of the effect indicates that the injection of electrons in the system can also come from local potential traps, like inhomogeneity of the sample or unintentional fabrication residue, which can vary considerably from place to place.

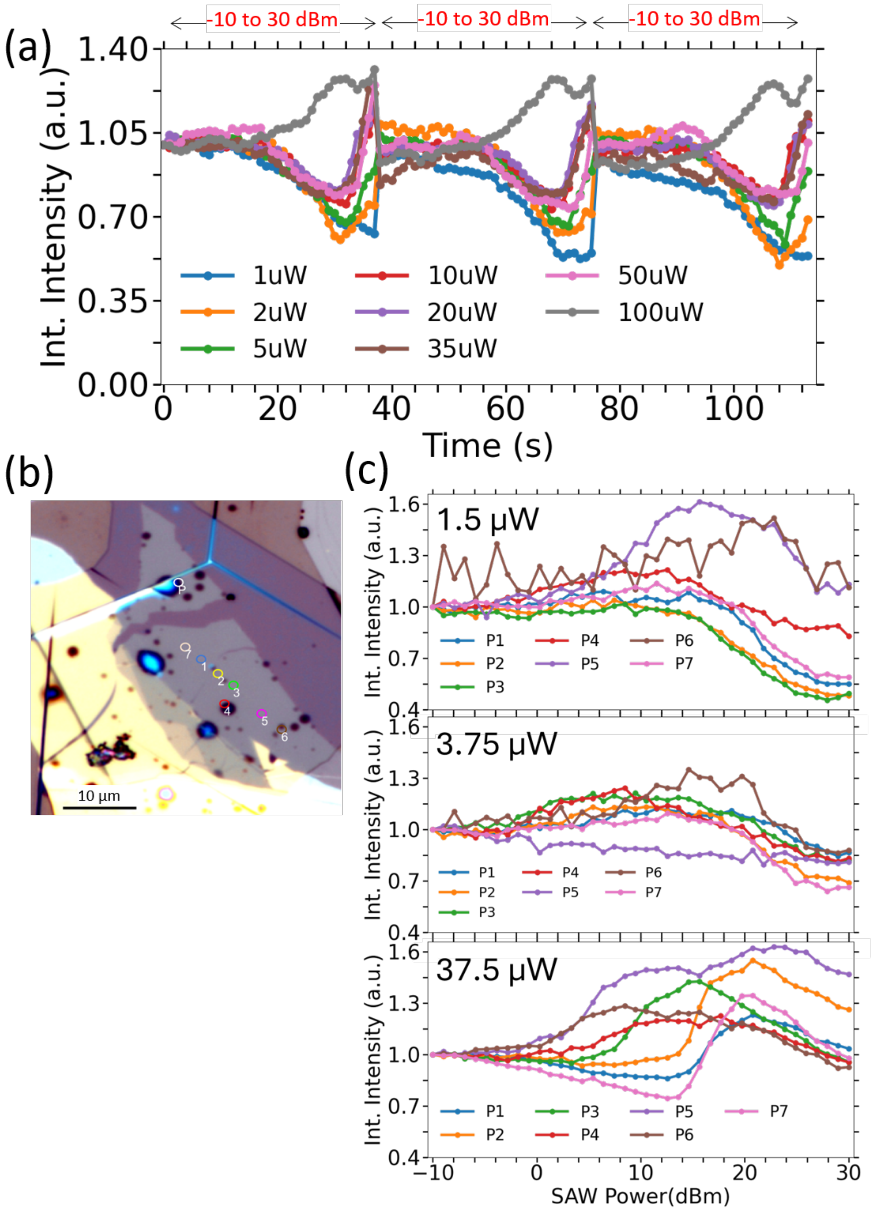

Figure S9: Measurements a function of time at room temperature. (a) Three cycles (one performed just after the other) of PL measurements where the SAW is increased from -10 to 30dBm in steps of 1 dBm. (b) Viewgraph of the sample indicating 7 positions where the measurements where taken. (c) Average (over the 3 cycles) PL intensity as a function the applied SAW power measured at the positions indicated in (b) at different laser power excitations.

Figure S10 presents the room temperature normalized PL intensity before the SAW is turned on for different laser excitation powers corresponding to the data presented in Figure 4(b) of the main text. It shows, as mentioned in the text, that as the laser is kept on the

sample for some time, the overall PL intensity decays in time due to photodoping. When the SAW is turned on and starts to take some carriers away from the laser generation spot, this doping can be partially reversed, which is the explanation for the increase in PL intensity observed in Figure S9.

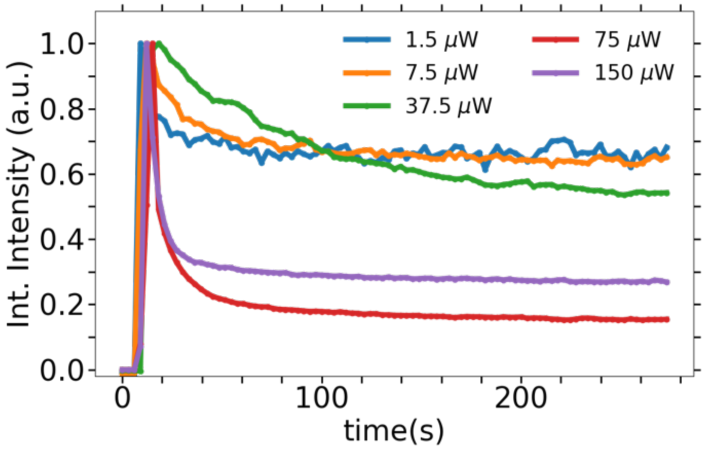

Figure S10: Normalized PL intensity of the hBN/WSe_2/hBN system measured at room temperature as a function of time, prior to the instant when the SAW is turned on.

Energy modulation with SAWs

The SAW modulates the excitonic properties of a TMD structure via the interaction with its propagating strain and its piezoelectric field. In some circumstances, for relatively high acoustic powers, this is reflected in energy shifts in the μ-PL spectrum of the excitonic emission lines.[13,14] Therefore, the acoustic modulation (ΔE_{SAW}) can be written as a combination of the energy shift induced by the strain and the piezoelectric field (Stark):[15]

$$\Delta E_{SAW} \;=\; \Delta E_{Stark} + \Delta E_{strain} = \frac{1}{2}\alpha_X E_x^2 + a_0 \frac{\delta V}{V} \tag{3}$$

where α_X is the in-plane exciton polarizability, E_x the in-plane component of the SAW piezoelectric field, a_0 the hydrostatic band gap deformation potential, and $\delta V/V$ is the

hydrostatic deformation.

Figure S11 presents finite element (FEM) simulations of the SAW strain and piezoelectric fields acting on the vdWH. The calculations were performed following the procedure described in reference,[13] i.e., taking into account the FEUDT dimensions and the radio-frequency reflection coefficient (S_{11}) measurement of the device. Figures S11(a) and (b) present the amplitude of E_x and $\delta V/V$ as a function of the applied rf power (P_{SAW}), respectively. Figure S11(c) shows the expected amplitude (modulus) of the energy modulation expected for the strain (ΔE_{strain}) and Stark (ΔE_{Stark}) components obtained using $\alpha_X = 10\text{meVcm}^2/\text{kV}^2$[16,17] and $a_0 = 35\text{meV}/\%$.[18]

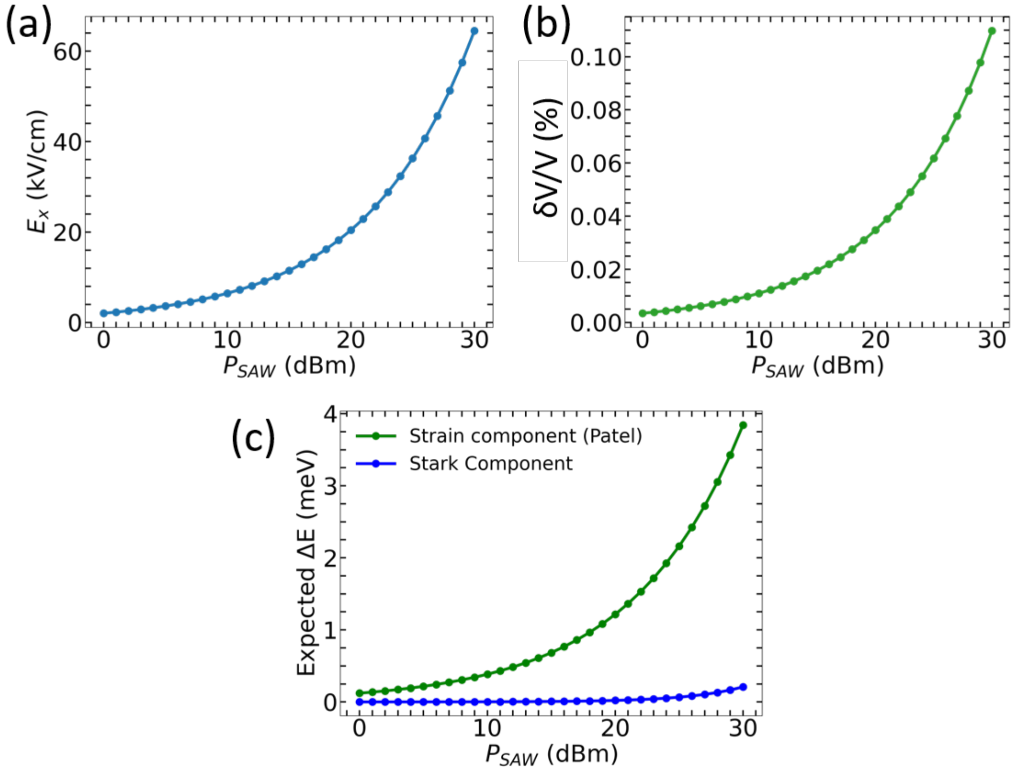

Figure S11: FEM simulations. (a) In-plane component (E_x) of the SAW piezoelectric field and (b) Hydrostatic deformation ($\delta V/V$) as a function of the applied rf power. (c) Expected energy modulation as a function of the SAW rf power calculated using the material exciton polarizability[16,17] and potential deformation.[18]

On the other hand, Figure S11(c) shows that the contribution of the strain component to the bandgap modulation of the structure is expected to be more relevant than the Stark

one due to the weak dielectric screening induced by the hBN. However, as observed in the 1.5μW laser power measurement shown in Figure 4(b) of the main text, we do not observe a shift in the PL emission of the vdWH. Figure S12 presents the FWHM of the neutral exciton (X^0) and trion (X^-) as a function of the SAW power extracted from this same set of measurements. Again, we do not observe a consistent linewidth broadening with the SAW power which would be an indication of an effective emission splitting due the compressive and tensile SAW strains. We, therefore, believe that the absence of pronounced strain effects in our measurements can be a combination of (*i*) the considerable difference between the magnitude of the expected modulation and the excitonic linewidths detected experimentally and (*ii*) a relaxed mechanical coupling between the vdWH and the $LiNbO_3$ substrate (in the sense that the $LiNbO_3$ strain is not fully transferred to the vdWH, as considered in the numerical simulations). The latter is reasonable since one of the main effects of using hBN is to decrease the mechanical coupling between the monolayers and the substrate, creating smoother structures and resulting in narrower linewidths.[19] This result shows that, as stated in the main text, the SAW piezoelectric field is the main responsible for the exciton dissociation dynamics described in this work.

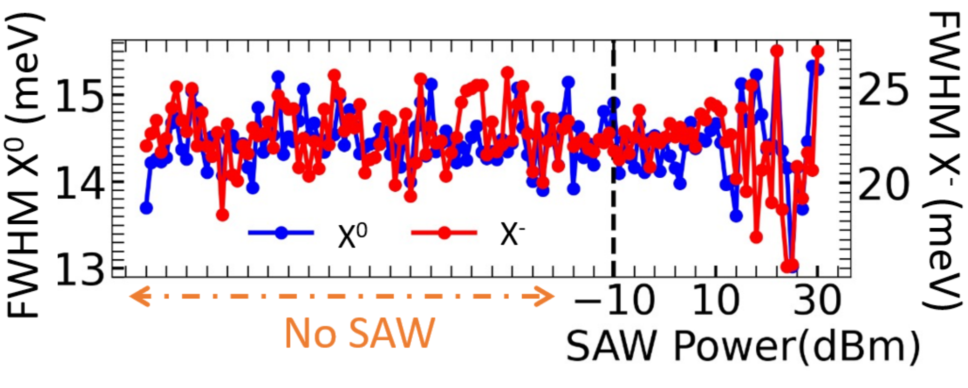

Figure S12: FWHM for the X^0 (blue dots, left vertical scale) and X^- (red dots, right vertical scale) emissions at room temperature before and after the SAW is turned on. The acoustic power is swept from -10 to 30 dBm during this measurement.

References

1. Cadore, A. R.; Rosa, B. L. T.; Paradisanos, I.; Mignuzzi, S.; Fazio, D. D.; Alexeev, E. M.; Dagkli, A.; Muench, J. E.; Kakavelakis, G.; Shinde, S. M.; Yoon, D.; Tongay, S.; Watanabe, K.; Taniguchi, T.; Lidorikis, E.; Goykhman, I.; Soavi, G.; Ferrari, A. C. Monolayer WS_2 electro- and photo-luminescence enhancement by TFSI treatment. *2D Mater.* **2024**, *11*, 025017.

2. Barbone, M.; Montblanch, A. R.-P.; Kara, D. M.; Palacios-Berraquero, C.; Cadore, A. R.; Fazio, D. D.; Pingault, B.; Mostaani, E.; Li, H.; Chen, B.; Watanabe, K.; Taniguchi, T.; Tongay, S.; Wang, G.; Ferrari, A. C.; Atatüre, M. Charge-tuneable biexciton complexes in monolayer WSe_2. *Nat. Commun.* **2018**, *9*, 3721.

3. Li, Z.; Wang, T.; Lu, Z.; Khatoniar, M.; Lian, Z.; Meng, Y.; Blei, M.; Taniguchi, T.; Watanabe, K.; McGill, S. A.; Tongay, S.; Menon, V. M.; Smirnov, D.; Shi, S. F. Direct Observation of Gate-Tunable Dark Trions in Monolayer WSe_2. *Nano Lett.* **2019**, *19*, 6886.

4. Li, Z.; Wang, T.; Lu, Z.; Jin, C.; Chen, Y.; Meng, Y.; Lian, Z.; Taniguchi, T.; Watanabe, K.; Zhang, S.; Smirnova, D.; Shi, S.-F. Revealing the biexciton and trion-exciton complexes in BN encapsulated WSe_2. *Nat. Commun.* **2018**, *9*, 3719.

5. Liu, E.; van Baren, J.; Lu, Z.; Altaiary, M. M.; Taniguchi, T.; Watanabe, K.; Smirnov, D.; Lui, C. H. Gate Tunable Dark Trions in Monolayer WSe_2. *Phys. Rev. Lett* **2019**, *123*, 027401.

6. Mostaani, E.; Szyniszewski, M.; Price, C. H.; Maezono, R.; Danovich, M.; Hunt, R. J.; Drummond, N. D.; Falko, V. I. Diffusion quantum Monte Carlo study of excitonic complexes in two-dimensional transition-metal dichalcogenides. *Phys. Rev. B* **2017**, *96*, 075431.

7. Zhou, Y.; Scuri, G.; Wild, D. S.; High, A. A.; Dibos, A.; Jauregui, L. A.; Shu, C.; Greve, K. D.; Pistunova, K.; Joe, A. Y.; Taniguchi, T.; Watanabe, K.; Kim, P.; Lukin, M. D.; Park, H. Probing dark excitons in atomically thin semiconductors via near-field coupling to surface plasmon polaritons. *Nature Nanotechnol.* **2017**, *12*, 856.

8. Wang, G.; Robert, C.; Glazov, M. M.; Cadiz, F.; Courtade, E.; Amand, T.; Lagarde, D.; Taniguchi, T.; Watanabe, K.; Urbaszek, B.; Marie, X. In-Plane Propagation of Light in Transition Metal Dichalcogenide Monolayers: Optical Selection Rules. *Phys. Rev. Lett* **2017**, *119*, 047401.

9. Park, K. D.; Jiang, T.; Clark, G.; Xu, X.; Raschker, M. B. Radiative control of dark excitons at room temperature by nano-optical antenna-tip Purcell effect. *Nature Nanotechnol.* **2018**, *13*, 59.

10. Li, Z.; Wang, T.; Jin, C.; Lu, Z.; Lian, Z.; Meng, Y.; Blei, M.; Gao, S.; Taniguchi, T.; Watanabe, K.; Ren, T.; Tongay, S.; Yang, L.; Smirnov, D.; Cao, T.; Shi, S.-F. Emerging photoluminescence from the dark-exciton phonon replica in monolayer WSe_2. *Nat. Commun.* **2019**, *10*, 2469.

11. Huang, J.; Hoang, T. B.; Mikkelsen, M. H. Probing the origin of excitonic states in monolayer WSe_2. *Sci. Rep.* **2016**, *6*, 22414.

12. Zhang, X.-X.; You, Y.; Zhao, S. Y. F.; Heinz, T. F. Experimental Evidence for Dark Excitons in Monolayer WSe_2. *Phys. Rev. Lett.* **2015**, *115*, 257403.

13. Scolfaro, D.; Finamor, M.; Trinchão, L.; Rosa, B. L. T.; Chavez, A.; Santos, P. V.; Iikawa, F.; Couto Jr., O. D. D. Acoustically Driven Stark Effect in Transition Metal Dichalcogenide Monolayers. *ACS Nano* **2021**, *15*, 15371.

14. Datta, K.; Li, Z.; Lyu, Z.; Deotare, P. B. Piezoelectric Modulation of Excitonic Properties in Monolayer WSe_2 under Strong Dielectric Screening. *ACS Nano* **2021**, *15*, 12334.

15. Couto, Jr., O. D. D.; Hey, R.; Santos, P. V. Spin dynamics in (110) GaAs quantum wells under surface acoustic waves. *Phys. Rev. B* **2008**, *78*, 153305.

16. Cavalcante, L. S. R.; da Costa, D. R.; Farias, G. A.; Reichman, D. R.; Chaves, A. Stark Shift of Excitons and Trions in Two-Dimensional Materials. *Phys. Rev. B* **2018**, *98*, 245309.

17. Massicotte, M.; Vialla, F.; Schmidt, P.; Lundeberg, M. B.; Latini, S.; Haastrup, S.; Danovich, M.; Davydovskaya, D.; Watanabe, K.; Taniguchi, T.; Fal'ko, V.; Thygesen, K. S.; Pedersen, T. G.; Koppens, F. H. Dissociation of Two-Dimensional Excitons in Monolayer WSe_2. *Nat. Commun.* **2018**, *9*, 1633.

18. Patel, S. D.; Parto, K.; Choquer, M.; Lewis, N.; Umezawa, S.; Hellman, L.; Polishchuk, D.; Moody, G. Surface Acoustic Wave Cavity Optomechanics with Atomically Thin h-BN and WSe_2 Single-Photon Emitters. *PRX Quantum* **2024**, *5*, 010330.

19. Cadiz, F.; Courtade, E.; Robert, C.; Wang, G.; Shen, Y.; Cai, H.; Taniguchi, T.; Watanabe, K.; Carrere, H.; Lagarde, D.; Manca, M.; Amand, T.; Renucci, P.; Tongay, S.; Marie, X.; ; Urbaszek, B. Excitonic Linewidth Approaching the Homogeneous Limit in MoS_2-Based van der Waals Heterostructures. *Phys. Rev. X* **2017**, *7*, 021026.
